# The LL-100 panel: 100 cell lines for blood cancer studies

**DOI:** 10.1038/s41598-019-44491-x

**Published:** 2019-06-03

**Authors:** Hilmar Quentmeier, Claudia Pommerenke, Wilhelm G. Dirks, Sonja Eberth, Max Koeppel, Roderick A. F. MacLeod, Stefan Nagel, Klaus Steube, Cord C. Uphoff, Hans G. Drexler

**Affiliations:** Leibniz-Institute DSMZ-German Collection of Microorganisms and Cell Cultures, Department of Human and Animal Cell Lines, Braunschweig, Germany

**Keywords:** Cancer models, Haematological cancer

## Abstract

For many years, immortalized cell lines have been used as model systems for cancer research. Cell line panels were established for basic research and drug development, but did not cover the full spectrum of leukemia and lymphoma. Therefore, we now developed a novel panel (LL-100), 100 cell lines covering 22 entities of human leukemia and lymphoma including T-cell, B-cell and myeloid malignancies. Importantly, all cell lines are unequivocally authenticated and assigned to the correct tissue. Cell line samples were proven to be free of mycoplasma and non-inherent virus contamination. Whole exome sequencing and RNA-sequencing of the 100 cell lines were conducted with a uniform methodology to complement existing data on these publicly available cell lines. We show that such comprehensive sequencing data can be used to find lymphoma-subtype-characteristic copy number aberrations, mRNA isoforms, transcription factor activities and expression patterns of NKL homeobox genes. These exemplary studies confirm that the novel LL-100 panel will be useful for understanding the function of oncogenes and tumor suppressor genes and to develop targeted therapies.

## Introduction

Human cancer cell lines form a renewable resource and are vital models for studying the cellular and molecular mechanisms underlying tumorigenesis as well as for anti-cancer drug screening^1,2^. In particular, leukemia-lymphoma (LL) cell lines serve as convenient *in vitro* tool due to their world-wide accessibility, straightforward manipulability and low culture costs, providing experimental models to address a multitude of questions in the field of LL biology^3^. Indeed, the scientific benefits of utilizing LL cell lines have definitely boosted our knowledge on a plethora of aspects of these diseases^4^. Importantly, many studies contoured our appreciation of the suitability of LL cell lines as model systems, replicating faithfully most features of the primary cells^5,6^.

The National Cancer Institute (NCI) tumor cell line panel (known as NCI-60 as 60 cancer cell lines were assembled) was developed in the 1980s as an *in vitro* drug discovery tool intended to supplant animal studies in drug screening (reviewed in^7^). This screening tool was quickly appreciated as an invaluable source of information about the mechanisms of growth inhibition and tumor cell cytotoxicity^7^. Later in the 2000s, the NCI-60 panel transitioned from a drug-discovery pipeline to a more general research tool in support of the cancer research community^7,8^. Another panel incorporating a reduced number of cell lines of particular interest which had been derived from several solid tumor types was established in Japan^9^. These two cell line panels did not aim at one single cancer category but were designed to represent a variety of different tumor entities. Nevertheless, these sets have provided the framework for the use of defined panels of cell lines at the same time as keeping with the information-rich character of screens^7^.

The majority of studies in the arena of LL focus on a narrow number of cell lines. We realized that there is a need for a reference panel specialized on LL cell lines to facilitate hypothesis-driven research efforts^10^. We have assembled a panel of 100 authenticated LL cell lines that reflects the heterogeneity of the entities under the umbrella category of LL. In addition to well-known and commonly analyzed cell lines, this invaluable and publicly available platform includes additional cell lines assigned unequivocally to the various entities but with specific characteristics. It is hoped that this focused LL-100 cell lines panel may enhance the current scientific momentum, helping to fully elucidate the underlying pathology of these LL malignancies and providing an important and unique resource for the testing of novel therapeutic agents.

Based on data of the human genome project, high-throughput methods have boosted the knowledge of processes in normal and malignant cells. The microarray technology showed for the first time simultaneous activities of thousands of genes and allowed the classification of tissues and diseases^11^. This approach is being steadily replaced by next generation sequencing technologies which comprise the sequencing of complete transcriptomes, exomes and whole genomes. These applications are used in cancer research to identify aberrations in the genome, deregulated and mutated genes, and alternative splicing. The obtained data are helpful to classify malignancies, to improve existing therapies, and to identify new targets for novel therapeutic approaches^12^. Here, we present transcriptome and exome sequencing data of a panel of 100 authenticated LL cell lines (LL-100) and selected examples of their utilization.

## Results and Discussion

### Sequencing of exomes and transcriptomes of the LL-100 panel

We performed whole exome sequencing (WES) and mRNA-sequencing (RNA-seq) on a panel of 100 LL cell lines representing 22 subtypes (Table 1). For exomic analyses over 10 million reads (2 × 151 bases) per sample were sequenced resulting in at least 50x coverage on a 60 MB exome size. RNA-seq yielded over 29 million (2 × 151 bases) reads per sample. Sequencing data have been deposited at ENA under the accession number PRJEB30297 for WES and PRJEB30312 for RNA-seq, respectively.

**Table 1:** The LL-100 panel.

Entity	Cell line	DSMZ ACC #	Selected aberrations
Pre-B-ALL	697	42	TCF3-PBX1
	KOPN-8	552	KMT2A-MLLT1
	NALM-6	128	
	REH	22	ETV6-RUNX1; RUNX1-PRDM7
	SEM	546	KMT2A-AFF1
B-NHL: Burkitt/B-ALL	BJAB	757	KMT2A-CLTC*
	DAUDI	78	t(8;14)(q24;q32)
	RAJI	319	IGH-MYC
	RAMOS	603	IGH-MYC
	VAL	586	t(8;14;18)(q24;q32;q21)
B-NHL: CLL/PLL	HG-3	765	
	JVM-3	18	
	JVM-13	19	
	MEC-1	497	R3HCC1L-HTRA1*
	PGA-1	766	
B-NHL: DLBCL ABC	NU-DHL-1	583	t(3;8)(p25;q24); t(14;18)(q32;q21)
	OCI-LY3	761	IGH-SPIB
	RI-1	585	t(4;8)(q22;q24); BCL2 amp
	U-2932	633	t(8;14)(q24;q32) in clone R2
	U-2946	795	IGH-MYC
B-NHL: DLBCL GC	DOHH-2	47	t(8;14;18)(q24;q32;q21)
	OCI-LY7	688	IGH-MYC
	OCI-LY19	528	t(14;18)(q32;q21)
	SU-DHL-4	495	EZH2 Y646S; IGH-BCL2
	SU-DHL-6	572	EZH2 Y646N; IGH-BCL2
	WSU-DLCL2	575	EZH2 Y646F
B-NHL: HCL	BONNA-12	150	
	HAIR-M	762	IGH-TCL1A*
	HC-1	301	
B-NHL: MCL	GRANTA-519	342	t(11;14)(q13;q32)
	JEKO-1	553	t(11;14)(q13;q32)
	JVM-2	12	t(11;14)(q13;q32)
	MINO	687	t(11;14)(q13;q32)
	REC-1	584	t(11;14)(q13;q32)
B-NHL: PEL	BC-3	679	
	BCBL-1	683	MYC amp
	CRO-AP2	48	
	CRO-AP5	215	
B-NHL: PMBL	U-2940	634	biallelic SOCS1 del
Multiple Myeloma/PCL	KMS-12-BM	551	t(11;14)(q13;q32)
	L-363	49	
	LP-1	41	IGH-WHSC1*
	OPM-2	50	IGH-WHSC1
	RPMI-8226	402	
	U-266	9	
Hodgkin Lymphoma	HDLM-2	17	
	KM-H2	8	CIITA-C15ORF65
	L-428	197	EZH2 Y646S
	L-1236	530	SOCS1 L150V; SOCS1 L162R
	SUP-HD1	574	
T-ALL/T-LL	CCRF-CEM	240	NKX2.5-BCL11B
	DND-41	525	TLX3-BCL11B
	HPB-ALL	483	t(5;14)(q35;q32); CBFB-MYLPF*
	JURKAT	282	
	MOLT-4	362	
	RPMI-8402	290	LMO1-TRD; SIL-TAL1
Mature T-Malignancy	DERL-7	524	
	HH	707	FOXK2-TP63*
	MOTN-1	559	TBL1XR1-TP63
NK Malignancy	KHYG-1	725	
	NK-92	488	
	YT	434	
ALCL	DEL	338	NPM1-ALK
	SR-786	369	NPM1-ALK
	SU-DHL-1	356	NPM1-ALK
	SUP-M2	509	NPM1-ALK
AML myelocytic	EOL-1	386	KMT2A PTD; FIP1L1-PDGFRA
	HL-60	3	
	KASUMI-1	220	RUNX1-RUNX1T1
	KG-1	14	FGFR1OP2-FGFR1
	NB-4	207	PML-RARA
	OCI-AML3	582	NPMcy type A, DNMT3A R882C
	SKNO-1	690	RUNX1-RUNX1T1
AML monocytic	ME-1	537	CBFB-MYH11
	MOLM-13	554	FLT3 ITD, KMT2A-MLLT3
	MONO-MAC-6	124	KMT2A-MLLT3; RUNX1-ATP8A2
	MUTZ-3	295	
	THP-1	16	CSNK2A1-DDX39B
	U-937	5	MLLT10-PICALM
AML erythroid	F-36P	543	
	HEL	11	JAK2 V617F
	OCI-M2	619	RUNX1-TSPEAR*
	TF-1	334	CBFA2T3-ABHD12*
AML megakaryocytic	CMK	392	JAK3 A572V
	ELF-153	693	
	M-07e	104	ANO7-DHDH*
	MEGAL	719	SET-NUP214
	MKPL-1	697	RBM6-CSF1R
	UT-7	137	
CML myeloid BC	EM-2	135	BCR-ABL1
	K-562	10	BCR-ABL1
	KCL-22	519	BCR-ABL1
	KU-812	378	BCR-ABL1
	LAMA-84	168	BCR-ABL1
	MOLM-20	591	KMT2A-SEPT11
CML lymphoid BC	BV-173	20	BCR-ABL1
	CML-T1	7	BCR-ABL1
	NALM-1	131	BCR-ABL1
	TK-6	723	BCR-ABL1; MAPK1-AIF1L*
MPN	SET-2	608	JAK2 V617F

Cell lines are available from the DSMZ cell lines bank (www.dsmz.de) which is a public cell line repository. The DSMZ is a non-profit research institute of the public Leibniz Association that is owned and subsidized by German federal and state governments.

Abbreviations: ALCL, anaplastic large cell lymphoma; ALL, acute lymphoblastic leukemia; AML, acute myeloid leukemia; amp, amplification; BC, blast crisis; B-NHL, B-Non Hodgkin lymphoma; CLL, chronic lymphocytic leukemia; CML, chronic myeloid leukemia; DLBCL ABC, diffuse large B cell lymphoma activated B-cell subtype; DLBCL GC, diffuse large B cell lymphoma germinal center subtype; HCL, hairy cell leukemia; ITD, internal tandem duplication; LL, lymphoblastic lymphoma; NK, natural killer; MCL, mantle cell lymphoma; MPN, myeloproliferative neoplasm; PCL, plasma cell leukemia; PEL, primary effusion lymphoma; PLL, prolymphocytic leukemia; PMBL, primary mediastinal B-cell lymphoma; PTD, partial tandem duplication. *Yet undescribed aberrations detected in this study by WES or RNA-seq analysis of the LL-100 panel.

Based on the analysis of WES and RNA-seq data we show the usefulness of the LL-100 panel for LL research in five exemplary studies.

### PEL and HL cell lines cluster separate from cell lines of other B-NHL entities

For many years, expression profiling has been applied to classify tumors including LL^11^. RNA-seq and microarray analyses show highly reproducible results with correlation between expression profiles^13^. We performed cluster analysis to test whether the two techniques yield comparable results also in the LL-100 panel. We analyzed gene expression of primary effusion lymphoma (PEL) cell lines and of cell lines from various other B-non Hodgkin lymphoma (B-NHL) entities as well as from Hodgkin lymphoma (HL).

**Figure 1:**
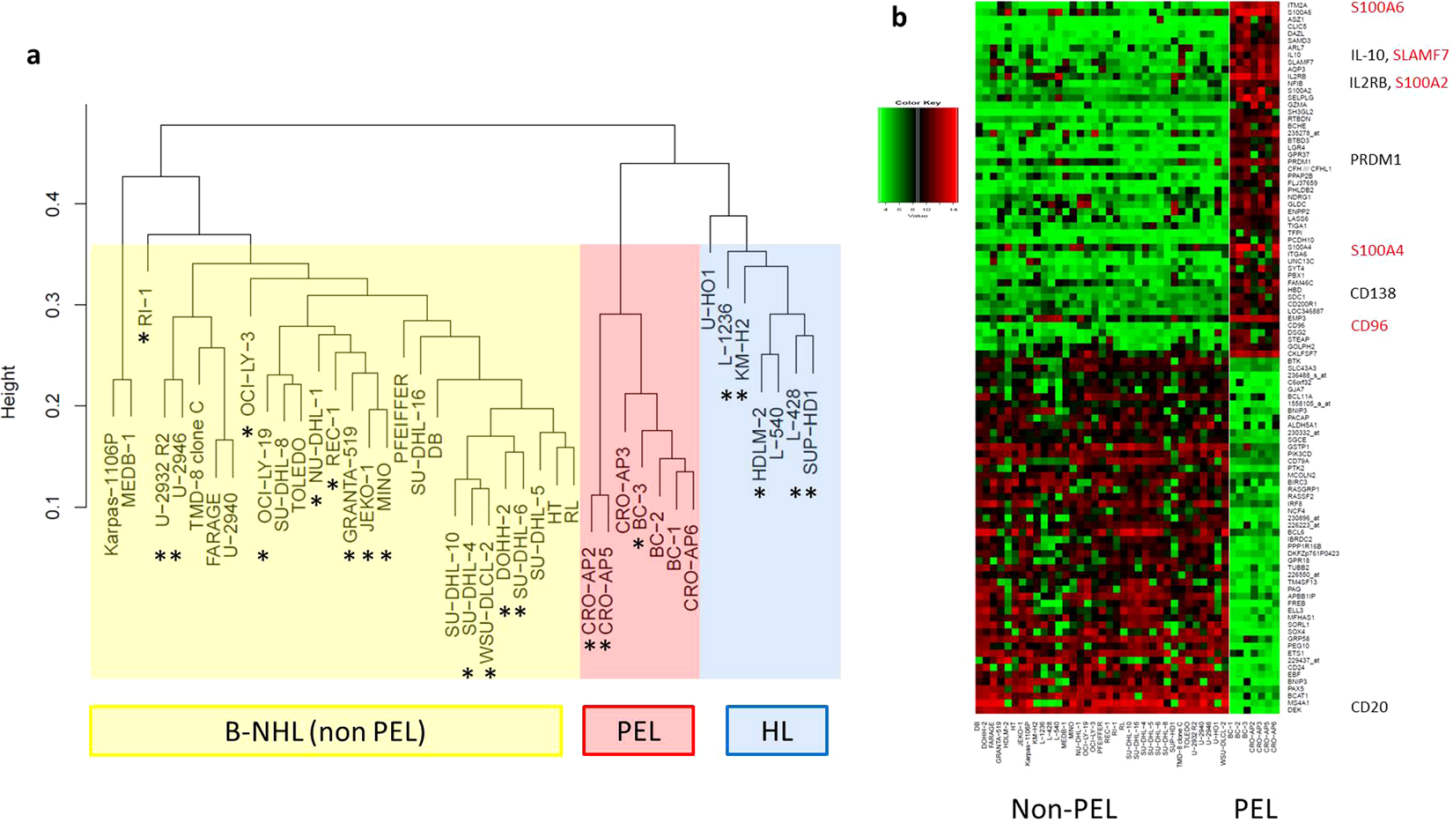
Microarray analysis of HL and B-NHL cell lines including PEL. (**a**) PEL cell lines (in red color) cluster separately from other B-NHL (in yellow color), but on the same arm as HL cell lines (in blue color). 30% of the most variant probe sets were taken for hierarchical clustering by average linkage. (**b**) PEL cell lines show tumor type-specific expression of genes including *SLAMF7*. For highest validity, the analysis was conducted with expression array data from LL-100 cell lines and additional cell lines. LL-100 cell lines are marked with an asterisk. Differentially expressed probe sets were filtered for one probe set per gene and top 50 positive and negative fold changes. Black: previously described PEL-specific genes; red: PEL-specific genes not described hitherto.

Unsupervised cluster analysis showed that all PEL cell lines grouped together, separate from cell lines derived from activated-B-cell-like (ABC) and germinal center (GC) diffuse large B-cell lymphoma (DLBCL), mantle cell lymphoma (MCL), primary mediastinal B-cell lymphoma (PMBL) and from cell lines derived from HL (Fig. 1a). Notably, PEL and HL cell lines clustered on one arm, separate from all cell lines representing the other B-NHL entities (Fig. 1a). Microarray and RNA-seq data yielded identical results, confirming the suitability of both techniques (Figs 1a, S1).

PEL and HL cell lines are characterized by a set of common up- and downregulated genes (Fig. S2). Prominent were expression of *CCND2* and the absence of B-cell markers in PEL and HL cell lines. *CD19, CD20 (MS4A1), CD24, CD79A* and *CD79B* were expressed in all tested lymphoma entities beside PEL and HL (Fig. S2). Absence, low or rare expression of these “early” B-cell markers in PEL and HL has been described for both primary lymphoma cells and cell lines^14–16^.

Highly expressed in PEL – but not in HL - were *CD138* (*SDC1*), *IL-10, IL2RB*, and *PRDM1*, all described as PEL-characteristic genes (Fig. 1b)^17–19^. Not reported hitherto was that PEL cells expressed *CD96, SLAMF7*, *S100A2, S100A4* and *S100A6* (Fig. 1b). RT-PCR, flow cytometry and Western blot analysis confirmed the PEL-associated expression of *CD138, PRDM1/*BLIMP1*, SLAMF7* and the three *S100A* family genes (Fig. S3a–c).

*PRDM1*/BLIMP1 is a master regulator of terminal B-cell differentiation. Originally described as repressor^20^, BLIMP1 can also enhance transcription of *SLAMF7* in multiple myeloma^21^ and of *IL-10* in type 1 regulatory T-cells^22^. Thus, coexpression of the three genes in PEL suggests a causal relationship between transcriptionally active *PRDM1* and the targets *SLAMF7* and *IL-10* also in this B-NHL entity. Independent of its regulation, the expression of *SLAMF7* in PEL is remarkable because a monoclonal antibody targeting SLAMF7 (elotuzumab) has recently been approved for treatment of patients with multiple myeloma^23^. RQ-PCR analysis showed that *SLAMF7* is comparably expressed in PEL and multiple myeloma cell lines (Fig. S4).

PEL is a rare, aggressive form of NHL, cells typically being infected with HHV-8^14^. With a median survival time of six months the prognosis for PEL patients is poor^24^. If our cell line results can be translated to primary tumor cells, PEL patients might benefit from targeted therapy with elotuzumab.

### Activities of hematopoietic transcription factors in leukemia-lymphoma cell lines

Numerous transcription factors (TF) regulate normal hematopoiesis and their activities are precisely controlled during hematopoietic stem cell self-renewal and their differentiation into the diverse blood cell lineages. Consequently, many of these TFs emerged as proto-oncogenes or tumor suppressors because deregulation of these TFs alters the cellular transcriptional program eventually impairing differentiation and thus fostering malignant transformation. Aberrant activities of TFs which can be caused by a variety of direct or indirect mutations and epigenetic alterations, are a hallmark of cancer, including hematological malignancies^25,26^.

**Figure 2:**
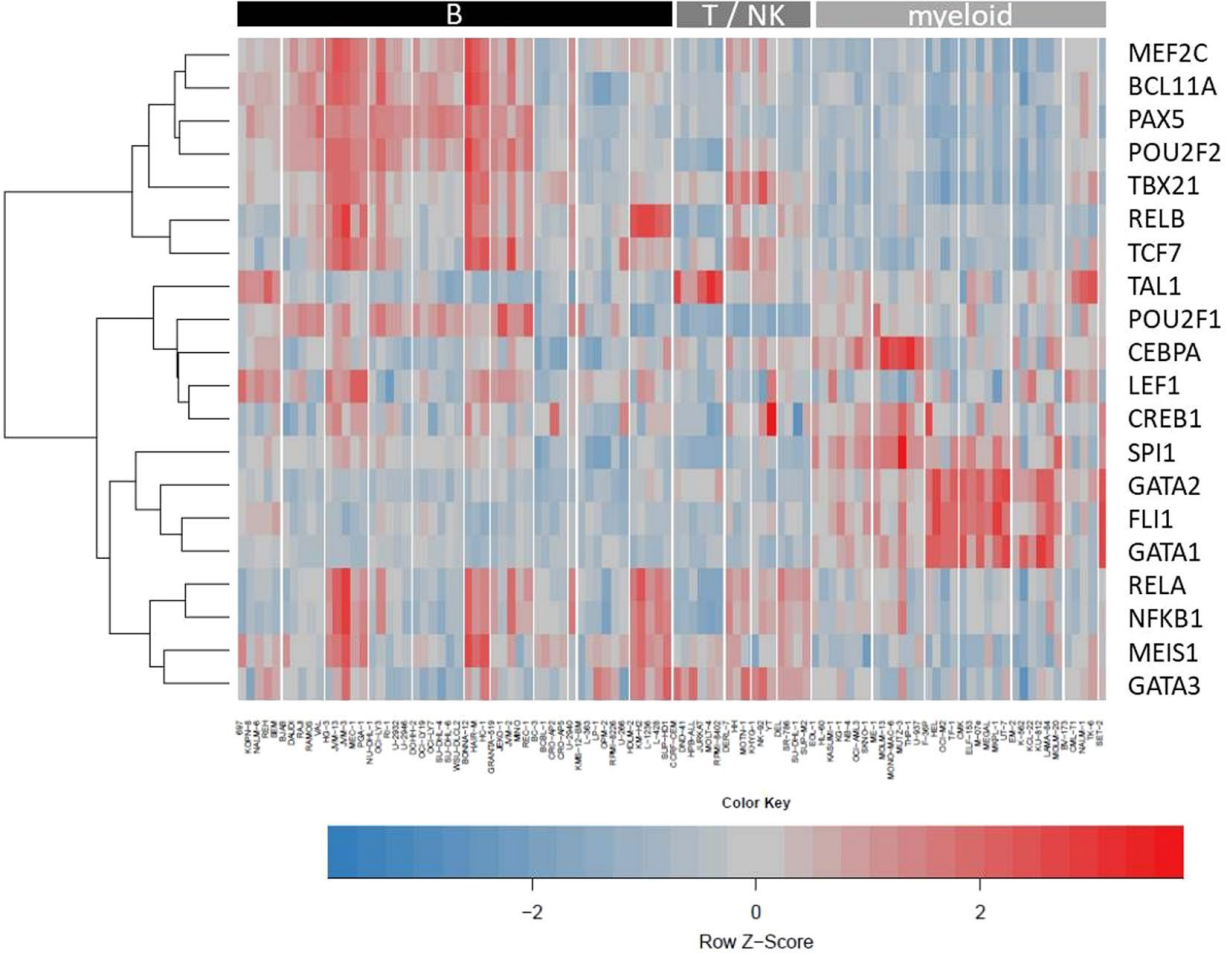
Activities of hematopoietic TFs across LL-100 cell lines according to their CTFR activity scores. Hierarchical clustering heatmap (distance: Euclidian; agglomeration method: complete; centered to row) of TF activities calculated via VIPER based on DoRothEA TF-interactions for each cell line from RNA-seq data. Each column represents a single cell line. Cell lines are grouped according to entities. The color code depicts the activity for each row-wise normalized CTFR. Bars on top of the heatmap indicate cellular origin of cell lines.

We aimed to analyze TF activities from TFs relevant for hematopoiesis across the LL-100 panel. Because the expression level of a TF itself barely gives information about its downstream activity^27^, activities of TFs were predicted via the expression levels of their direct target genes. TF activities were estimated via so-called consensus TF regulons (CTFRs) which have been defined recently by Garcia-Alonso *et al*. on the basis of diverse sources for human TF-target interactions^28,29^. For each cell line from the LL-100 panel relative TF activities were computed from RNA-seq data applying DoRothEA (Discriminant Regulon Expression Analysis) for CTFRs from 289 single TFs (Table S1). From these 289 TFs we selected 20 TFs based on their known role in hematopoiesis. The activities of the respective CTFRs in the LL-100 cell lines are represented in Fig. 2.

Obviously, activity patterns of several TFs within the cell lines mirror their cell of origin: PAX5 and OCT-2 (encoded by *POU2F2*) are critical for B-cell development^30^. Accordingly, the CTFRs of these TFs showed strong activity in cell lines from B-cell derived malignancies but were inactive in myeloid-derived leukemias (Fig. 2). Other TF activities reflect the differentiation status of their respective normal counterparts: the strong activity of the CTFRs from GATA1 and GATA2 was highly specific for the cell lines from erythroid and megakaryocytic AML, CML and in cell line SET-2 (myeloproliferative neoplasm) (Fig. 2), which is in line with the role of GATA1 and GATA2 in the differentiation of erythroid-megakaryocytic progenitors where alterations in their dosages are related to transformation^31^.

Other CTFR activities indicate the mutation status of hematopoietic TFs in specific entities: C/EBPα is a TF relevant for granulopoiesis and AML patients frequently show inactivating mutations of C/EBPα impairing final differentiation of the cells^32^. Accordingly, the activity of the CEBPA-CTFR was diminished in erythroid and megakaryocytic AML cell lines compared to myelocytic and monocytic AML cell lines (Fig. 2).

Another subset of TF activities is characteristic for specific leukemia or lymphoma entities: TAL1 impairs T-cell differentiation and is a master oncogene in T-ALL^33^. Accordingly, T-ALL cell lines showed the strongest activity of the TAL1-CTFR (Fig. 2).

The lymphocyte specific TF LEF1 was primarily active in pre-B-ALL and CLL/PLL cell lines but rather inactive in T-ALL and ALCL cell lines (Fig. 2), which is in line with the current literature^34–36^. In addition, the LEF1-CTFR activity was moderately upregulated in MCL cell lines and some BL and GC-DLBCL cell lines (Fig. 2). This activity pattern seems to reflect the situation in patients because upregulation of LEF1 in subsets of B-NHL patients has been reported before^37–39^.

For 9 of the 20 hematopoietic TFs we observed a moderate positive correlation between gene expression levels of the TFs and their corresponding CTFR activities (Table 2, Fig. S5). Best correlations were detected for PU.1 (encoded by *SPI1*) and GATA2. However, in general hematopoietic TF activities determined via CTFRs did hardly correlate with gene expression levels of the TFs (Fig. S6). For example *TAL1* expression was rather weak in cell lines from CML in blast crisis (Fig. S5), but the TAL1-CTFR activity was increased in these cell lines (Fig. 2). On the other hand *TAL1* expression was detected in several AML cell lines on a comparable level to T-ALL cell lines (Fig. S5), but activity of the TAL1-CTFR was low in AML cell lines (Fig. 2). This underpins that upregulation of a TF alone is not sufficient to regulate its target genes. In some cases a defined CTFR (e.g. from TAL1) might also be regulated by further transcriptional activators or repressors.

**Table 2:** Correlation analysis between CTFR activity and gene expression levels.

TF gene name	mean CTFR activity score	mean TF expression [logCPM]	Spearman correlation coefficient
BCL11A	2.68	3.34	0.32
CEBPA	0.16	1.23	0.28
CREB1	−0.51	6.19	−0.13
FLI1	−0.56	6.33	0.31
GATA1	−1.24	−1.31	0.55
GATA2	0.11	0.57	0.74
GATA3	−1.79	1.09	0.66
LEF1	−0.22	3.54	0.05
MEF2C	3.06	5.49	0.37
MEIS1	−0.81	1.38	0.02
NFKB1	−1.37	6.50	0.55
PAX5	2.95	2.82	0.71
POU2F1	0.06	5.91	0.27
POU2F2	2.77	4.37	0.64
RELA	−0.49	6.44	0.06
RELB	2.12	4.82	0.70
SPI1	−0.78	3.31	0.75
TAL1	0.98	−0.55	0.28
TBX21	2.52	−0.64	0.55
TCF7	2.11	1.38	0.43

Correlation between CTFR activity and gene expression levels from 20 hematopoietic TFs across the LL-100 panel. CTFR activity scores were computed via VIPER based on DoRothEA interactions.

In summary, activity scores of CTFRs are much more informative concerning the role of a TF than its transcript levels alone. We show that transcriptional activities in LL-100 cell lines mirror the lineage origin of hematologic malignancies for a set of specific TFs (e.g. PAX5). Other TFs (e.g. GATA1) reflect the differentiation status of the respective normal counterpart and a third group of TFs (e.g. TAL1) depicts aberrant activities highly characteristic for specific entities. In general, TF activities across all studied cell lines did rarely correlate with their gene expression levels. Thus, analyses of CTFR activities from RNA-seq data are a suitable tool to measure and evaluate the relevance of a specific TF in hematological cell lines.

### Aberrant NKL homeobox gene activities in lymphoid malignancies

Homeobox genes encode TFs which show basic impacts in developmental processes including embryonal development and cell differentiation in the adult. Therefore, deregulation of homeobox genes generates developmental disturbances or cancer^40^. These genes are classified according to differences in their conserved homeobox and ordered into classes and subclasses^41^. The NKL subclass comprises 48 members which are involved in fundamental differentiation processes like *NKX2-1* in that of the lung and the thyroid, and *NKX2-5* in the heart^42,43^. Normal expression patterns of nine NKL homeobox genes in early hematopoiesis and subsequent lymphocyte development have been identified and termed hematopoietic NKL-code^44,45^. According to this code, T-cells silence all NKL homeobox genes during their thymic development while mature NK-cells maintain expression of *MSX1* and mature B-cells *HHEX* or *NKX6-3*^44–46^. Alterations of the NKL-code may underlie the generation of particular hematopoietic malignancies. According to this notion, 24 NKL homeobox genes are reported to date for aberrant activity in T-cell acute lymphoblastic leukemia (T-ALL), mediating differentiation arrest and transformation^44,47,48^.

**Figure 3:**
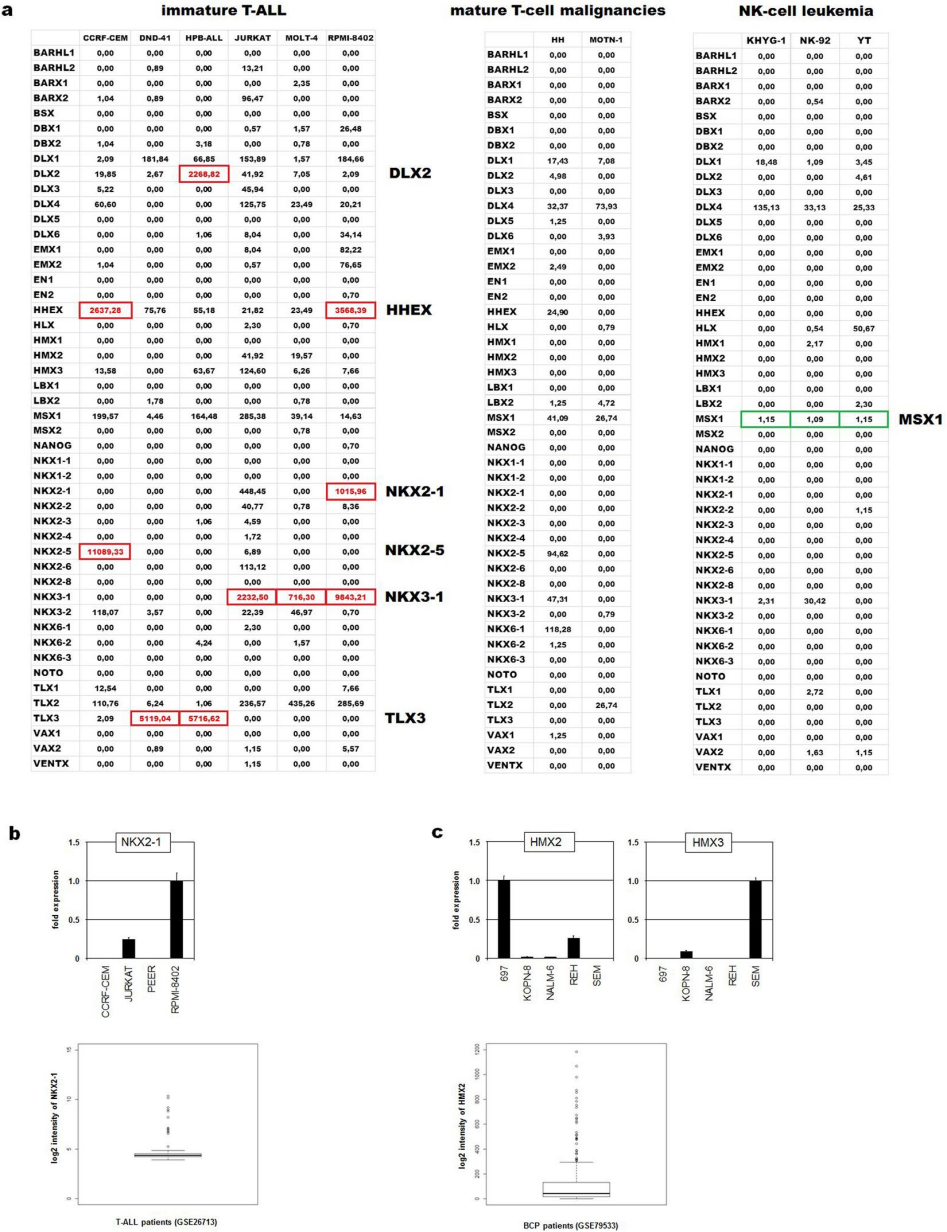
Expression data of 48 NKL homeobox genes in selected cell lines. (**a**) The indicated tables show expression levels of 48 NKL homeobox genes in six immature T-ALL cell lines (left), in two cell lines derived from mature T-cell malignancies (middle), and three NK-cell leukemia cell lines (right). The listed values are normalized counts (reads) obtained by DESeq2 calculation. Overexpressed or downregulated gene activities are highlighted by red or green boxes, respectively. (**b**) RQ-PCR analysis of selected T-ALL cell lines shows elevated *NKX2-1* expression levels in RPMI-8402 cells (above). The indicated box plot (below) shows *NKX2-1* expression levels in 117 T-ALL patient samples obtained from GSE26713. Outliers indicate samples with *NKX2-1* overexpression. (**c**) RQ-PCR analysis in selected BCP-ALL cell lines shows elevated expression levels of *HMX2* in 697 cells and of *HMX3* in SEM cells (above). The indicated box plot (below) shows *HMX2* expression levels in 229 BCP-ALL patient samples obtained from GSE79533. Outliers indicate samples with *HMX2* overexpression.

Using the LL-100 transcriptome dataset we here screened NKL homeobox gene activities in cell lines and show some results for selected lymphoid entities. To discriminate active and inactive genes we have set a cutoff at 500 normalized counts. Accordingly, aberrant activation of particular subclass members was detected in immature T-ALL but not in mature T-cell lines (Fig. 3a). This finding supported the observation that NKL homeo-oncogenes provoke an arrest in differentiation which plays a role in immature thymocytes but obviously not in mature T-cells. Furthermore, *MSX1* is an oncogene in T-ALL and a tumor suppressor in NK-cells^46,49^, showing accordingly reduced activity in NK-cell leukemia cell lines (Fig. 3a). Thus, these data confirm published deregulated NKL homeobox genes including *MSX1, NKX2-5, NKX3-1* and *TLX3* in T-cell and NK-cell leukemia. Moreover, our RNA-seq data indicated elevated *NKX2-1* expression in T-ALL cell line RPMI-8402 (Fig. 3a). Aberrant activation of *NKX2-1* has been identified in T-ALL patients by chromosomal translocation, representing thus an additional clinically relevant oncogene^50^. Subsequent RQ-PCR analysis confirmed *NKX2-1* activity in this cell line (Fig. 3b). Of note, chromosomal and genomic analyses indicated absence of a translocation or an amplification targeting *NKX2-1* in RPMI-8402 cells (data not shown). Therefore, this cell line may represent a model to examine alternative upstream and novel downstream factors of *NKX2-1* in T-ALL.

In normal B-cell development NKL homeobox genes HHEX and NKX6-3 are the only subclass members active in developing naïve and germinal center B-cells, and in mature memory B-cells and plasma cells while B-cell progenitor (BCP) cells additionally express *HLX* and *MSX1*^44,45^. Our data show that malignant BCP-ALL cell lines lack activity of *HLX*, *MSX1* and *NKX6-3* (except SEM) (Fig. S7), showing fundamental changes in the normal expression pattern of NKL homeobox genes. Furthermore, three of five cell lines aberrantly expressed *HMX2* or *HMX3*. The activity of these genes was confirmed by RQ-PCR in the indicated cell lines (Fig. 3c). Moreover, *HMX2* overexpression was detected in 13% of 229 BCP-ALL patients by analysis of public dataset GSE79533 (Fig. 3c), supporting the clinical relevance of this finding. Thus, *HMX2* (and *HMX3*) may represent NKL homeobox genes primarily deregulated in this type of B-cell malignancy, serving as diagnostic marker and/or therapeutic target.

In DLBCL cell lines we detected silencing of *HHEX* (in OCI-LY7 and RI-1) and *NKX6-3* (except DOHH-2) and aberrant activation of *HLX* (NU-DHL-1) and *NKX3-1* (OCI-LY3) (Fig. S7). Of note, these data did not show significant differences between ABC- and GC-DLBCL cell lines, suggesting that NKL homeobox genes do not play a role in the discrimination of these disease subtypes. Surprisingly, PEL and MM cell lines (except RPMI-8226 expressing BARX2) demonstrated complete absence of NKL homeobox gene activity (Fig. S7). Therefore, NKL subclass members may operate as basic tumor suppressors in these particular B-cell lymphoma types. The malignant cells of both PEL and MM are derived from mature B-cells suggesting that in final stages of development NKL homeobox genes lose their oncogenic potential. Finally, the lack of B-cell specific NKL homeobox gene activity in PEL is in accordance with reported downregulation of general B-cell factors as indicated above (Fig. S2)^15^. Together, deregulation of NKL homeobox genes in B-cell malignancies is more important than hitherto expected. The identified cell lines may serve as models to investigate the role of these genes in the indicated tumor entities. Thus, the LL-100 datasets allow the identification of cell line models for the examination of deregulated NKL homeobox genes in particular disease entities. The expression patterns of this fundamental gene subclass in cell lines reflect the situation observed in normal lymphocytes and in primary tumor cells, highlighting the significance of these cell line data for cancer research.

### Copy number alterations and their effect on gene expression in DLBCL

DLBCL shows a high degree of genetic diversity with unique molecular patterns including varying occurrences of copy number alterations (CNAs), resembling the different states of B-cell maturation they derive from and which is also reflected by the diverse clinical outcome^51–54^.

**Figure 4:**
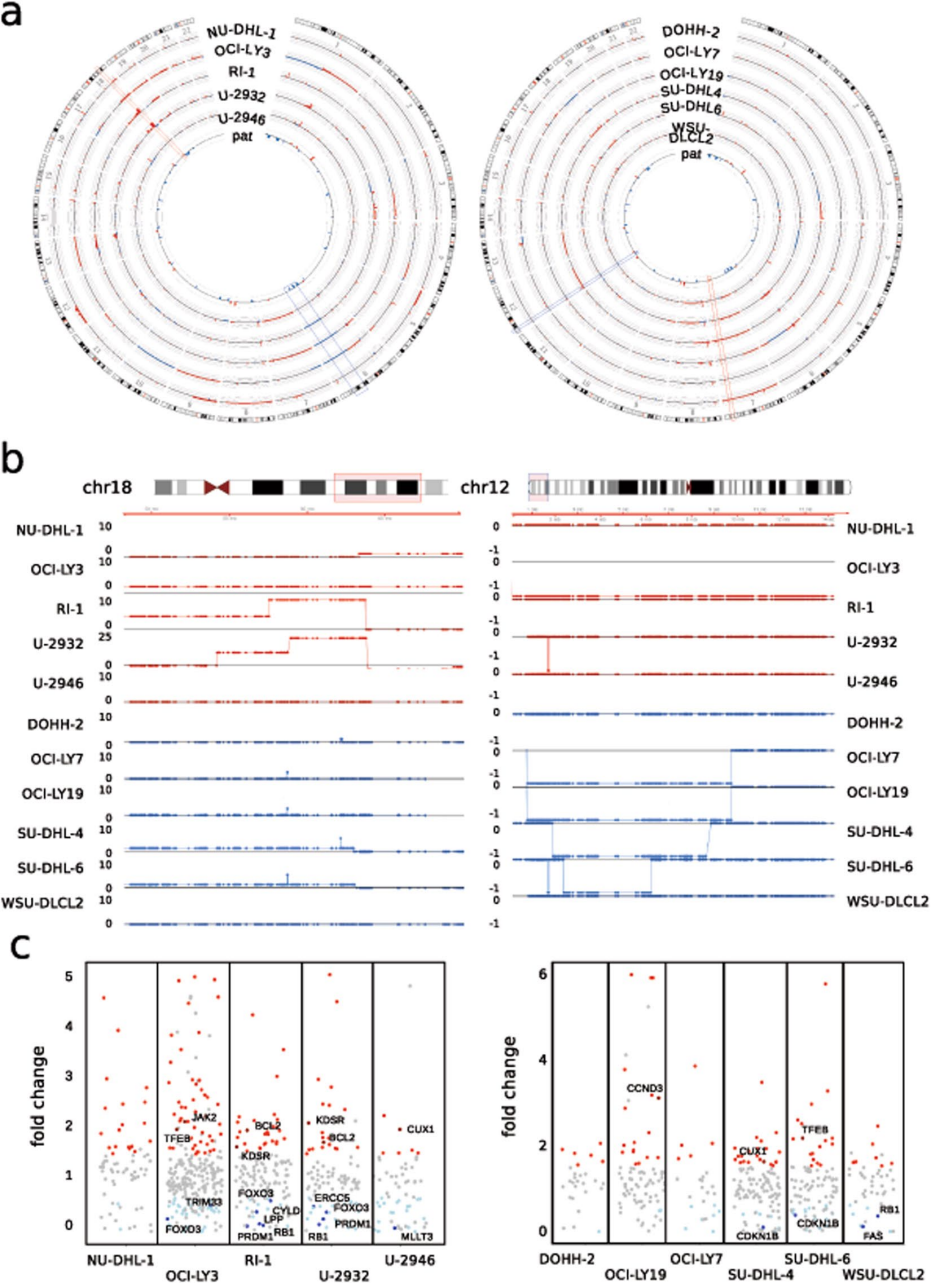
Copy Number Alterations (CNAs) and their impact on gene expression in DLBCL cell lines. (**a**) Circos plots depict the landscape of CNAs in cell lines of DLBCL subtypes ABC-DLBCL (left) and GC-DLBCL (right). Chromosome 1–22 are shown with the copy number gains in red and deletions in blue, the cell lines analyzed are indicated. The inner ring displays significant CNAs identifed in patients from Chapuy *et al*.^55^. Red and blue boxes highlight individual regions with gains or deletions, respectively that are common to patients and cell lines. (**b**) Examples of DLBCL-subtype specific alterations. Region of 18q22-q23 preferentially amplified in ABC-DLBCL (left) and 6q deletion in the GC-subtype (right) are shown for the indicated cell lines. DLBCL cell lines of the ABC-subtype are shown in red, those of GC-origin in blue, the respective copy number is shown on the left. Each dot resembles one exon from WES, the black line denotes 2n – normal copy number state. (**c**) Expression of COSMIC cancer genes affected by CNAs in ABC- and GC-DLBCL cell lines, left and right, respectively. All genes with expression change >1.5 are shown either in red or light blue, labeled are those that have been described as deregulated in a patient cohort by Chapuy *et al*.^55^.

To evaluate such alterations and to test if differences in the molecular subtypes are maintained in culture, we used WES data generated in our study to call CNAs in DLBCL derived cell lines. In ABC and GC DLBCL, we identified on average 157 (+/−29) and 129 (+/−12) CNAs, respectively, with a size >10 kb (Fig. 4a and Table 3). While amplification of both arms of chr7 occurs frequently in both subtypes, certain events like the 6q-deletion seem to occur more often in the ABC-subtype. Intending to compare the identified events to primary tumors, we took a recently published set of significantly recurrent CNAs in DLBCL which described a total of 45 recurring focal events (14 amplifications and 31 deletions) in 304 patients^55^. Of those CNAs, we find 38 (84%) in at least one cell line, including all focal amplifications (Fig. 4a and Table S2).

**Table 3:** Chromosomal gains and losses in ABC and GC DLBCL.

ABC subtype	Gains	Deletions	Total events
NU-DHL-1	109	20	129
OCI-LY3	139	44	183
RI-1	133	27	160
U-2932	150	37	187
U-2946	94	32	126
**GC subtype**	**Gains**	**Deletions**	**Total events**
DOHH-2	94	14	108
OCI-LY7	104	22	126
OCI-LY19	100	28	128
SU-DHL-4	123	21	144
SU-DHL-6	110	29	139
WSU-DLCL2	95	32	127

Cell lines were grouped according to ABC- or GC- subtype, total number of CNAs, and gains and losses. CNAs were called with control-FREEC using the B-lymphoblastoid cell line NC-NC as normal control. Neighbouring alterations with identical copy number were fused and CNAs <10 kb were omitted.

Similarly, of the 20 arm-level alterations described, 10 out of 18 amplifications and both recurrent deletions are present in one or more cell lines (Table S2). Of particular interest are subtype specific alterations, possibly reflecting different mutational processes during tumor development. Therefore, we assessed such specific events by integrating another set of ABC- or GC-related CNAs^56^ and found 83% of patient-derived events in our cell lines (15 out of 18; Table S2). In addition to the previously observed 6q-deletion, we could confirm a preferentially occurring gain of 18q22-q23 in ABC-DLBCL cell lines (3/5 ABC-DLBCL cell lines and 0/6 cell lines of the GC-subtype). Also, we find the deletion of the far end of 1p36 exclusively in 4/6 GC-DLBCL cell lines (Fig. 4b).

While CNAs can serve as diagnostic markers, their main impact results from associated changes in gene expression. We therefore compared the expression of all affected genes in a DLBCL-subtype specific manner, identifying around 20,000 genes to be affected by CNAs across all cell lines. Although we observe little overall changes for the majority of genes, several outliers are present in each cell line (Fig. S8). To identify CNA-deregulated genes important for disease progression, we filtered for those genes included in the COSMIC Cancer Gene Census database^57^. Depending on the cell line, we find 25 (OCI-LY7) to 252 (OCI-LY3) of the COSMIC genes located in regions of CNA (Table S3), with 10 (DOHH-2) to 121 (OCI-LY3) showing changes in expression >1.5, concordant with the respective CN change (Fig. 4c). Several of these genes have been confirmed deregulated in patient derived RNA-seq data^55^, which we also find associated with the corresponding CNA, e.g. *BCL2* and *KDSR* on chr18. Interestingly, we also find *PRDM1* and *FOXO3* to show reduced expression in ABC-DLBCL cell lines harboring a deletion of 6q21. This deletion has been described to be ABC-subtype related in an earlier study^53^. Nevertheless, these authors did not identify potential tumor-relevant genes in this deleted region^53^.

In summary, we exemplarily identified CNAs in cell lines derived from both DLBCL-subtypes and characterized the associated expressional changes. We find a high degree of similarity towards data from primary tumors and highlighted which cancer-relevant genes become deregulated in the individual cell lines.

This analysis (i) characterizes CNAs in cell lines of the two major DLBCL-subtypes and shows how they recapitulate recurring events from patients, (ii) allows the identification of those genes that are deregulated by CNAs and likely have a disease-relevant function and (iii) by doing so enables the selection of appropriate models for further molecular research related to DLBCL. Furthermore, we believe that this kind of analysis is applicable to other entities and that thereby valuable models for those entities can be obtained.

### Tissue-specific RNA isoforms

Allowing different combinations of exons, alternative splicing leads to the production of multiple mRNA isoforms of the same gene, often resulting in proteins of different functionality^58^. More than 90% of human genes are affected by alternative splicing^59,60^. Tissue-specific splice factors together with ubiquitious RNA binding factors cooperate to generate tissue-specific RNA isoforms^59^. The existence of different promoters can also lead to different N-terminal RNA variants.

**Figure 5:**
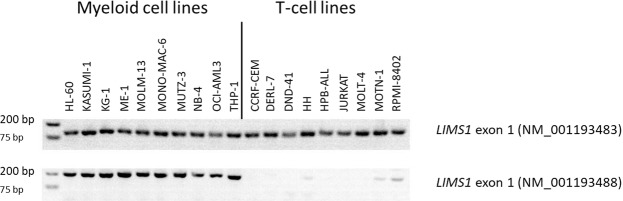
Expression of N-terminal *LIMS1* exons in myeloid and T-cell lines. RT-PCR analysis reveals expression of *LIMS1* (NM_001193488) exon 1 in myeloid cell lines only, expression of *LIMS1* (NM_001193483) exon 1 in myeloid cell lines and in T-cell lines. Data were confirmed in a second validation cohort (Fig. S10).

The RNA-seq data of the LL-100 panel allowed us to find RNA isoforms that specify different hematopoetic lineages. Bioinformatic analysis identified genes with tissue-specific exons, e.g. *LIMS1* in myeloid vs T-cell lines (Fig. S9). Two N-terminal variants of *LIMS1* were expressed in myeloid cell lines, only one of them in T-cell lines (Fig. S9). These results were confirmed by RT-PCR and validated with a second cell lines cohort (Figs 5, S10). Altogether, data from 18 AML and from 17 T-ALL cell lines revealed that the two groups could be distinguished on the basis of *LIMS1* exon 1 expression (NM_001193488) with a sensitivity of 1 and a specificity of 1.

In sum, our data show that RNA-seq analysis allows detection of cell lines from different lineages on the basis of alternatively expressed exons.

## Conclusion

One goal of personalized medicine in cancer medicine is the development of targeted therapies aiming to reverse detrimental effects of mutated or deregulated genes. The costs of sequencing technologies will presumably soon be low enough to allow routine diagnostics detecting genetic alterations for classification of the patient’s tumor and determining treatment strategies.

Immortalized tumor cell lines have been used for a long time to understand the molecular and cellular function of mutated genes and to develop new drugs. However, the cell line panels established hitherto did not represent most forms of leukemia and lymphoma^7,9^. Thus, the NCI-60 human cell line panel developed for use in drug development comprises sixty human cancer cell lines derived from nine different tissues^7^. Only six cell lines (CCRF-CEM, HL-60, K-562, MOLT-4, RPMI-8226, SR-786) represent tumors of the blood.

Covering 22 leukemia and lymphoma entities we present the novel LL-100 panel, 100 cell lines for use in basic research and drug development. The selected cell lines of this panel are authenticated and free of contamination by mycoplasma or non-inherent viruses. Furthermore, the methods of RNA- and DNA isolation and sequencing are identical in all cell lines. Therefore, this dataset allows comparative studies without methodical impact. We performed WES and RNA-seq analysis for all 100 cell lines. In exemplary studies, we show that lymphoma entities can be identified by gene expression analysis and splice variant analysis. WES analysis documented that copy number aberrations in DLBCL cell lines reflects the situation in primary tumor cells and may lead to the identification of potential oncogenes. RNA-seq analysis identified tumor entity-specific activities of CTFRs, demonstrating the usefulness of cell lines as model systems for transcription factor research. Finally, RNA-seq analysis specified aberrant activities of NKL homeobox genes.

All data and cell lines are publicly available. As demonstrated exemplarily in this study, the sequencing data can be used for various approaches. We hope that the novel LL-100 panel described here will stimulate many studies in the field of leukemia and lymphoma research.

## Methods

### Cell lines

Cell lines were taken from the stock of the cell lines bank (Leibniz Institute DSMZ – German Collection of Microorganisms and Cell Cultures). Cell lines were authenticated by DNA profiling and cytogenetics. Detailed references and cultivation protocols have been described previously^3^.

### RNA-sequencing analysis

Total RNA was extracted via miRNeasy Mini Kit (Qiagen, Hilden, Germany) including DNase digestion. Library preparation and sequencing steps were commissioned to GATC Biotech (Cologne, Germany). The GATC pipeline included the production of strand-specific (fr-first strand) mRNA libraries, quality control via Applied Biosystems Fragment Analyzer and Nanodrop, concentration measurement via Qubit fluorometer. The libraries were sequenced on Illumina HiSeq2500 (2 × 151 cycles, paired end run, 8 bp dual indices) with >29 million reads per sample and deposited at ENA (PRJEB30312). Reads were trimmed via fastq-mcf (ea-utils 1.04.807). Reads were quality controlled via FastQC (www.bioinformatics.babraham.ac.uk/projects/fastqc). Reads were aligned by STAR (2.5.3a)^61^ to the Gencode Homo sapiens genome (v26) and converted/sorted via samtools (0.1.19)^62^. Counting the reads to each gene was done via HTSeq-count python script (0.8.0)^63^. Data was processed and analyzed in the R/Bioconductor environment (3.3.2/3.3, www.bioconductor.org). Normalization, estimation of dispersions, and testing for differentially expressed genes based on a test assuming negative binomial data distribution was computed via DESeq2^64^.

Differential isoforms between given cell line groups were detected by JunctionSeq (JunctionSeq_1.4.0)^65^.

### Whole exome sequencing analysis

DNA was isolated with the High Pure PCR Template Preparation Kit (Roche Diagnostics, Mannheim, Germany). Library preparation (Agilent SureSelect Human All Exon V6, 60 MB) and sequencing steps (2 × 151 bp + 8 bp barcoding, HiSeqX) were commissioned to Genewiz (Leipzig, Germany) and deposited at ENA (PRJEB30297). Insert lengths were aimed to be higher than 250 bp in order to increase coverage and uniformity in coding regions^66^.

Reads were aligned by STAR (2.5.3a)^61^ to the human gencode genome (v26). Subsequently, alignment files were processed (samtools 0.1.19), duplicates removed (picard 2.9.2, www.broadinstitute.github.io/picard/), and variants called via GATK tools (3.7, Haplotypecaller)^67^ and overlapping VarScan (v2.4.3)^68^ results. Mutation effects were annotated via Ensembl VEP (release-84, GRCh38)^69^. Data were processed and analyzed in the R/Bioconductor environment (3.3.2/3.3). Overlapping single nucleotide variations via Haplotypecaller and VarScan were filtered for >=20 quality, >=10 depth, >=0.2 allele frequency, <0.01 MAF, missense/frameshift/stop gained mutations.

For identifying copy number alterations (CNA) in DLBCL cell lines control-FREEC^70^ (v11.0) was applied on the duplicate-clean alignment files with NC-NC as normal cell control. Neighboring regions with identical CN in the disperse whole exome data were fused to one region and CN regions below 10 kb were omitted and visualized via circos^71^ (0.67–7). Individual regions and genes of interest were plotted with the R/bioconductor packages ggplot2 (3.1.0) and Gviz (1.22.3).

### Expression array analysis

Profiling of gene expression was commissioned to the Genome Analytics Research Group, Helmholtz Centre for Infection Research, Braunschweig, Germany. 500 ng total RNA were used for biotin labelling according to the 3′ IVT Express Kit (Affymetrix, Santa Clara, CA, USA). 7.5 µg of biotinylated cDNA were fragmented and placed in a hybridization cocktail containing four biotinylated hybridization controls (BioB, BioC, BioD, and Cre). Samples were hybridized to an identical lot of Affymetrix GeneChip HG-U133 Plus 2.0 for 16 h at 45 °C. Steps for washing and SA-PE staining were processed on the fluidics station 450 using the recommended FS450 protocol (Affymetrix). Image analysis was performed on GCS3000 Scanner and GCOS1.2 Software Suite (Affymetrix). For data analysis spot intensities were RMA-background corrected and quantile normalized. Data processing was done via R/Bioconductor (3.2.2) using limma and affy packages^72,73^. Differentially expressed genes between PEL and Non-PEL cell lines were identified via moderated t-statistics (R, limma) setting p < 0.05 and log fold changes >1. For hierarchical cluster analysis (average linkage) 30% of the most varying probe sets served as basis for the distance matrix (1-correlation) and visualised via the R package hdust.

Public expression profiling datasets used in this study were generated by U133 Plus 2.0 gene chips from Affymetrix and obtained from Gene Expression Omnibus (GEO, www.ncbi.nlm.nih.gov). This type of profiling gene chip contains 37 probes of the 48 known human NKL homeobox genes, excluding HMX3. We analyzed datasets GSE26713 and GSE79533 for T-ALL and BCP-ALL patients, respectively. Expression values were given as boxplots using R-packages. Outliers were interpreted as samples showing aberrant overexpression.

### Analysis of TF activities

Relative TF activities were computed from RNA-seq data with DoRothEA v2 via VIPER^74^ for CTFRs with a TF-target score of high confidence (A), likely confidence (B), and medium confidence (C)^29^. For this, gene expression data were prepared similarly as described^28^: (i) lowly expressed genes were discarded, defined as those with an average CPM < 1 (counts per million) and prior count of 3; (ii) data were TMM (trimmed mean of M-values) normalized using calcNormFactors function described in limma; (iii) fitted log2 CPM were extracted and finally tested for transcriptional activity via VIPER in R/Bioconductor (3.4.4/3.6). Transcriptional activity and gene expression heatmaps were vizualised via gplots (R-package).

### Quantitative real-time PCR analysis and detection of splice variants

RNA was prepared using the RNeasy Mini kit (Qiagen). For mRNA quantification, reverse transcription was performed using the SuperScript II reverse transcriptase kit (Invitrogen, Karlsruhe, Germany). PCR was performed on a 7500 Applied Biosystems (Darmstadt, Germany). TaqMan probes (Applied Biosystems) were used to quantify human *CCND2* (Hs00153380_m1) *CD20/MS4A1* (Hs00544819_m1), *CD96* (Hs00175524_m1), CD138 (Hs00896424_g1), *HMX2* (Hs01394375_m1), *HMX3* (Hs01392772_m1), *IL-10* (Hs00961622_m1), *NKX2-1* (Hs00163037_m1), *PRDM1* (Hs00153357_m1), *S100A2* (Hs00195582_m1), *S100A4* (Hs00243202_m1), *S100A6* (Hs00170953_m1) and *SLAMF7* (Hs00900280_m1) using *TBP* as endogenous control. Relative expression levels were calculated using the ΔΔCt method.

Primers and RT-PCR conditions for detection of splice variants of *LIMS1, RABGAP1L* and *TFEC* are shown in Table S4.

### Cell surface marker analysis

For immunophenotyping, cells were washed and incubated for 30 min at 4 °C with antibodies (Ab) against CD319/SLAMF7 (#331802, BioLegend, Koblenz, Germany), CD138 Ab (#551902, Becton Dickinson, Heidelberg, Germany) and appropriate isotype controls (BD Biosciences). Subsequently, cells were treated with FITC conjugated anti-mouse secondary Ab (Biozol, Eching, Germany) and propidium iodide (PI) (Sigma-Aldrich). Labeled cells were analyzed on a FACSCalibur (BD Biosciences) using CellQuest Pro software.

### Western blot analysis

Samples were prepared as described previously^75^. Anti BLIMP1 (#9115), CCND2 (#3741S), S100A4 (#13018S), S100A6 (#13162S) Abs were purchased from Cell Signaling (New England Biolabs, Frankfurt, Germany). Anti GAPDH (ab8245) and anti S100A2 (ab 109494) Abs were from Abcam (Cambridge, UK). Specific bands on nitrocellulose membranes were visualized with the biotin/streptavidin-horseradish peroxidase system (GE Healthcare, Little Chalfont, UK) in combination with the “Renaissance Western Blot Chemoluminescence Reagent” protocol (Perkin Elmer, Waltham, MA, USA).

## Supplementary information

Dataset 1

Dataset 2

Dataset 3

Dataset 4

Dataset 5

## Data Availability

Sequencing data have been deposited at ENA under the accession number PRJEB30297 for WES and PRJEB30312 for RNA-seq, respectively. The ArrayExpress accession IDs are E-MTAB-7722 (WES) and E-MTAB-7721 (RNA-seq).

## References

[1] Barretina J (2012). The Cancer Cell Line Encyclopedia enables predictive modelling of anticancer drug sensitivity. Nature.

[2] Haverty PM (2016). Reproducible pharmacogenomic profiling of cancer cell line panels. Nature.

[3] Drexler, H. G. Guide to Leukemia-Lymphoma Cell Lines. 2nd Edition Braunschweig, eBook on CD (2010).

[4] MacLeod RA (2008). Human leukemia and lymphoma cell lines as models and resources. Curr. Med. Chem..

[5] Ross DT (2000). Systematic variation in gene expression patterns in human cancer cell lines. Nat. Genet..

[6] Sandberg R, Ernberg I (2005). Assessment of tumor characteristic gene expression in cell lines using a tissue similarity index (TSI). Proc. Natl. Acad. Sci. USA.

[7] Shoemaker RH (2006). The NCI60 human tumour cell line anticancer drug screen. Nat. Rev. Cancer.

[8] Abaan OD (2013). The exomes of the NCI-60 panel: a genomic resource for cancer biology and systems pharmacology. Cancer Res..

[9] Nakatsu N (2005). Chemosensitivity profile of cancer cell lines and identification of genes determining chemosensitivity by an integrated bioinformational approach using cDNA arrays. Mol. Cancer Ther..

[10] Drexler HG, MacLeod RA (2003). Leukemia-lymphoma cell lines as model systems for hematopoietic research. Ann. Med..

[11] Golub TR, Slonim DK, Lander ES (1999). Molecular classification of cancer: class discovery and class prediction by gene expression monitoring. Science.

[12] Cieślik M, Chinnaiyan AM (2018). Cancer transcriptome profiling at the junction of clinical translation. Nat. Rev. Genet..

[13] Zhao S (2014). Comparison of RNA-seq and microarray in transcriptome profiling of activated T cells. Plos One.

[14] Cesarman E, Knowles DM (1999). The role of Kaposi´s sarcoma-associated herpesvirus (KSHV HHV-8) in lymphoproliferative diseases. Semin. Cancer Biol..

[15] Carbone A (2010). Understanding pathogenetic aspects and clinical presentation of primary effusion lymphoma through its derived cell lines. AIDS.

[16] Tzankov A (2003). Expression of B-cell markers in classical Hodgkin lymphoma: a tissue microarray analysis of 330 cases. Mod. Pathol..

[17] Jones KD (1999). Involvement of interleukin-10 (IL-10) and viral IL-6 in the spontaneous growth of Kaposi´s sarcoma herpesvirus-associated infected primary effusion lymphoma cells. Blood.

[18] Jenner RG (2003). Kaposi’s sarcoma-associated herpesvirus-infected primary effusion lymphoma has a plasma cell gene expression profile. Proc. Natl. Acad. Sci. USA.

[19] Chadburn A (2008). Immunophenotypic analysis of the Kaposi sarcoma herpesvirus (KSHV; HHV-8)-infected B cells in HIV+ multicentric Castleman disease (MCD). Histopathology.

[20] Yu J (2000). Transcriptional repression by Blimp-1 (PRDI-BF1) involves recruitment of histone deacetylase. Mol. Cell. Biol..

[21] Kim JR, Mathew SO, Mathew PA (2016). Blimp-1/PRDM1 regulates the transcription of human CS1 (SLAMF7) gene in NK and B cells. Immunobiology.

[22] Montes de Oca M (2016). Blimp-1-dependent IL-10 production by Tr1 cells regulates TNF-mediated tissue pathology. PLOS Pathogens.

[23] Touzeau C, Moreau P, Dumontet C (2017). Monoclonal antibody therapy in multiple myeloma. Leukemia.

[24] Chen YB, Rahemtullah A, Hochberg E (2007). Primary effusion lymphoma. The Oncologist.

[25] Bradner JE, Hnisz D, Young RA (2017). Transcriptional addiction in cancer. Cell.

[26] Crans HN, Sakamoto KM (2001). Transcription factors and translocations in lymphoid and myeloid leukemia. Leukemia.

[27] Falco MM (2016). The pan-cancer pathological landscape. Sci. Rep..

[28] Garcia-Alonso L (2018). Transcription factor activities enhance markers of drug sensitivity in cancer. Cancer Res..

[29] Garcia-Alonso, L. *et al*. Benchmark and integration of resources for the estimation of human transcription factor activities. *bioRxiv*, 10.1101/337915 (preprint) (2018).

[30] Matthias P, Rolink AG (2005). Transcriptional networks in developing and mature B cells. Nat. Rev. Immunol..

[31] Shimizu R, Yamamoto M (2016). GATA-related hematologic disorders. Exp. Hematol..

[32] Koschmieder S (2005). Role of transcription factors C/EBPα and PU.1 in normal hematopoiesis and leukemia. Int. J. Hematol..

[33] Sanda T, Leong WZ (2017). TAL1 as a master oncogenic transcription factor in T-cell acute lymphoblastic leukemia. Exp. Hematol..

[34] Hassler MR (2016). Insights into the pathogenesis of anaplastic large-cell lymphoma through genome-wide DNA methylation profiling. Cell Reports.

[35] Kühnl A (2011). Overexpression of LEF1 predicts unfavorable outcome in adult patients with B-precursor acute lymphoblastic leukemia. Blood.

[36] Gutierrez A (2010). Inactivation of LEF1 in T-cell acute lymphoblastic leukemia. Blood.

[37] Walther N (2013). Aberrant lymphocyte enhancer-binding factor 1 expression is characteristic for sporadic Burkitt’s lymphoma. Am. J. Pathol..

[38] O’Malley DP, Lee JP, Bellizzi AM (2017). Expression of LEF1 in mantle cell lymphoma. Ann. Diagn. Pathol..

[39] Cubedo E (2012). Identification of LMO2 transcriptome and interactome in diffuse large B-cell lymphoma. Blood.

[40] Abate-Shen C (2002). Deregulated homeobox gene expression in cancer: cause or consequence?. Nat. Rev. Cancer.

[41] Holland PW, Booth HA, Bruford EA (2007). Classification and nomenclature of all human homeobox genes. BMC Biol..

[42] Lazzaro D (1991). The transcription factor TTF-1 is expressed at the onset of thyroid and lung morphogenesis and in restricted regions of the foetal brain. Development.

[43] Komuro I, Izumo S (1993). Csx: a murine homeobox-containing gene specifically expressed in the developing heart. Proc. Natl. Acad. Sci. USA.

[44] Nagel S (2017). NKL homeobox gene activities in hematopoietic stem cells, T-cell development and T-cell leukemia. PLOS One.

[45] Nagel S (2018). NKL heomeobox gene activities in B-cell development and lymphomas. PLOS One.

[46] Nagel S (2017). NKL homeobox gene MSX1 acts like a tumor suppressor in NK-cell leukemia. Oncotarget.

[47] Villarese P (2018). TCRα rearrangements identify a subgroup of NKL-deregulated adult T-ALLs associated with favorable outcome. Leukemia.

[48] Ferrando AA (2002). Gene expression signatures define novel oncogenic pathways in T cell acute lymphoblastic leukemia. Cancer Cell.

[49] Nagel S (2015). Repressed BMP signaling reactivates NKL homeobox gene MSX1 in a T-ALL subset. Leuk. Lymphoma.

[50] Homminga I (2011). Integrated transcript and genome analyses reveal NKX2-1 and MEF2C as potential oncogenes in T cell acute lymphoblastic leukemia. Cancer Cell.

[51] Basso K, Dalla-Favera R (2015). Germinal centres and B cell lymphomagenesis. Nat. Rev. Immunol..

[52] Staudt LM (2005). The biology of human lymphoid malignancies revealed by gene expression profiling. Adv. Immunol..

[53] Lenz G (2008). Molecular subtypes of diffuse large B-cell lymphoma arise by distinct genetic pathways. Proc. Natl. Acad. Sci. USA.

[54] Reddy A (2017). Genetic and functional drivers of diffuse large B cell lymphoma. Cell.

[55] Chapuy B (2018). Molecular subtypes of diffuse large B cell lymphoma are associated with distinct pathogenic mechanisms and outcomes. Nat. Med..

[56] Karube K (2018). Integrating genomic alterations in diffuse large B-cell lymphoma identifies new relevant pathways and potential therapeutic targets. Leukemia.

[57] Futreal PA (2004). A census of human cancer genes. Nat. Rev. Cancer.

[58] Badr E, El Hefnawi M, Heath LS (2016). Computational identification of tissue-specific splicing regulatory elements in human genes from RNA-seq data. PLOS One.

[59] Wang ET (2008). Alternative isoform regulation in human tissue transcriptomes. Nature.

[60] Chen M, Manley JL (2009). Mechanisms of alternative splicing regulation: insights from molecular and genomics approaches. Nat. Rev. Mol. Cell. Biol..

[61] Dobin A (2013). STAR: ultrafast universal RNA-seq aligner. Bioinformatics.

[62] Li H (2009). The sequence alignment/map format and SAMtools. Bioinformatics.

[63] Anders S, Pyl PT, Huber W (2015). HTSeq—a Python framework to work with high-throughput sequencing data. Bioinformatics.

[64] Anders S, Huber W (2010). Differential expression analysis for sequence count data. Genome Biol..

[65] Hartley SW, Mullikin JC (2016). Detection and visualization of differential splicing in RNA-seq data with JunctionSeq. Nucleic Acids Res..

[66] Pommerenke C (2016). Enhanced whole exome sequencing by higher DNA insert lengths. BMC Genomics.

[67] McKenna A (2010). The genome analysis toolkit: a MapReduce framework for analyzing next-generation DNA sequencing data. Genome Res..

[68] Koboldt DC (2012). VarScan 2: somatic mutation and copy number alteration discovery in cancer by exome sequencing. Genome Res..

[69] McLaren W (2010). Deriving the consequences of genomic variants with the Ensembl API and SNP effect predictor. Bioinformatics.

[70] Boeva V (2012). Control-FREEC: a tool for assessing copy number and allelic content using next-generation sequencing data. Bioinformatics.

[71] Krzywinski M (2009). Circos: an information aesthetic for comparative genomics. Genome Res..

[72] Ritchie ME (2015). Limma powers differential expression analyses for RNA-sequencing and microarray studies. Nucleic Acids Res..

[73] Gautier L (2004). Affy-analysis of Affymetrix GeneChip data at the probe level. Bioinformatics.

[74] Alvarez JJ (2016). Functional characterization of somatic mutations in cancer using network-based inference of protein activity. Nat. Genet..

[75] Quentmeier H (2009). SET-NUP214 fusion in acute myeloid leukemia- and T-cell acute lymphoblastic leukemia-derived cell lines. J. Hematol. Oncol..

