# Supplementary material for: The LL-100 panel: 100 cell lines for blood cancer studies
Source: Sci Rep. 2019 Jun 3;9:8218. doi: 10.1038/s41598-019-44491-x (PMC6547646; doi:10.1038/s41598-019-44491-x)
Supplement: Supplementary file 1 — Dataset 1 [file 41598_2019_44491_MOESM1_ESM.pdf]

The LL-100 panel: 100 cell lines for blood cancer studies

Running title: LL-100

Hilmar Quentmeier,* Claudia Pommerenke, Wilhelm G Dirks, Sonja Eberth, Max Koeppel, Roderick AF MacLeod, Stefan Nagel, Klaus Steube, Cord C Uphoff, Hans G Drexler

Leibniz-Institute DSMZ-German Collection of Microorganisms and Cell Cultures, Department of Human and Animal Cell Lines, Braunschweig, Germany

Email addresses:

HQ: hqu@dsmz.de; CP: cpo14@dsmz.de; WGD: wdi@dsmz.de; SE: seb14@dsmz.de; MK: mko16@dsmz.de; RAFM: rafmacleod@gmail.com; SN: sna@dsmz.de; KS: kst@dsmz.de; CCU: cup@dsmz.de; HGD: hdr@dsmz.de.

*Correspondence to: Dr. Hilmar Quentmeier

Inhoffenstr. 7B, 38124 Braunschweig, Germany

e-mail: hqu@dsmz.de

phone: ++49-531-2616-165

FAX: ++49-531-2616-150

Keywords: alternative splicing, cell lines, leukemia, lymphoma, PEL, SLAMF7, whole exome sequencing, RNA-seq

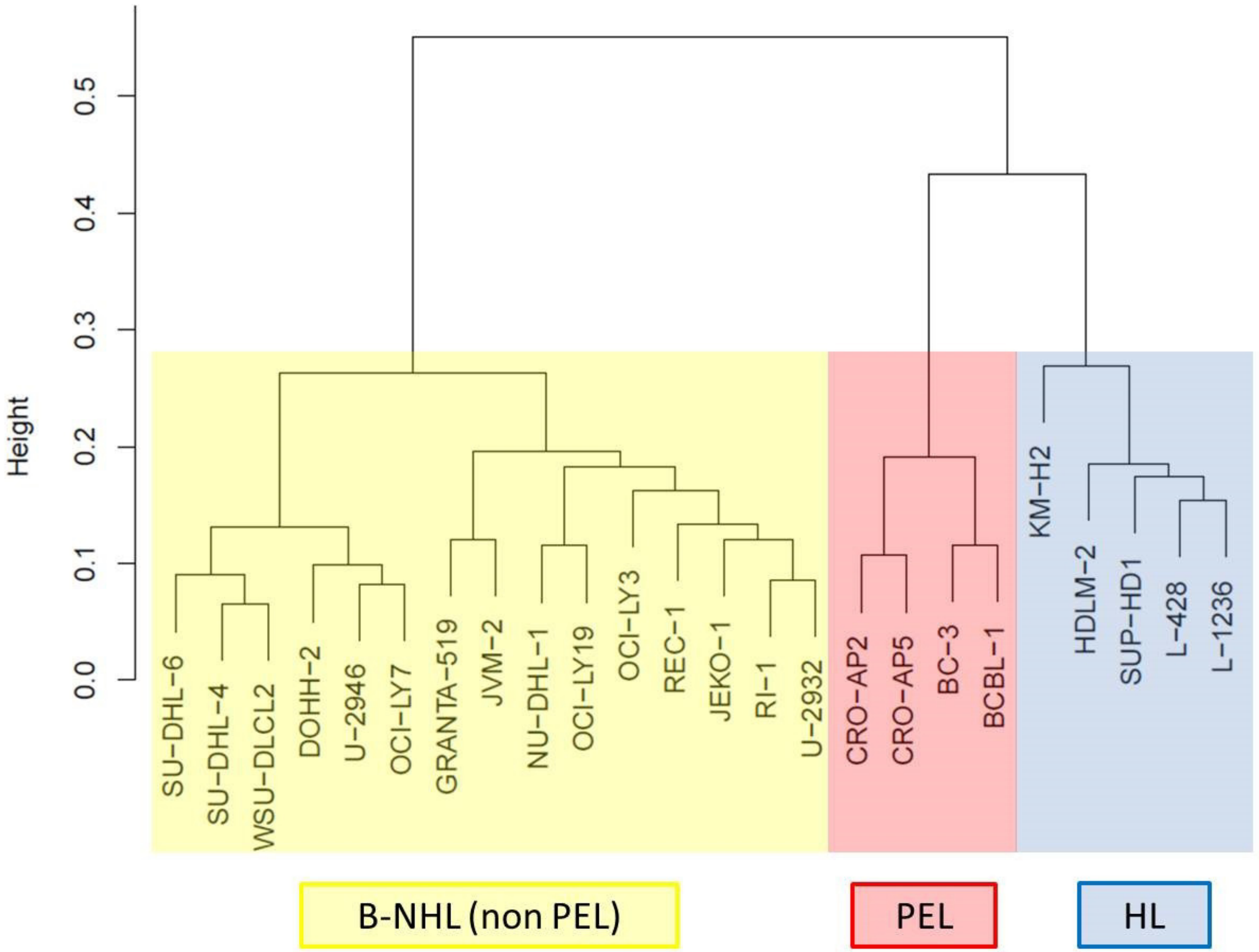

Supplementary Figure 1. RNA-seq-based expression analysis of HL and B-NHL cell lines including PEL. PEL cell lines (in red color) cluster separately from other B-NHL (in yellow color), but on the same arm as HL cell lines (in blue color), confirming the clustering by mircroarray analysis.

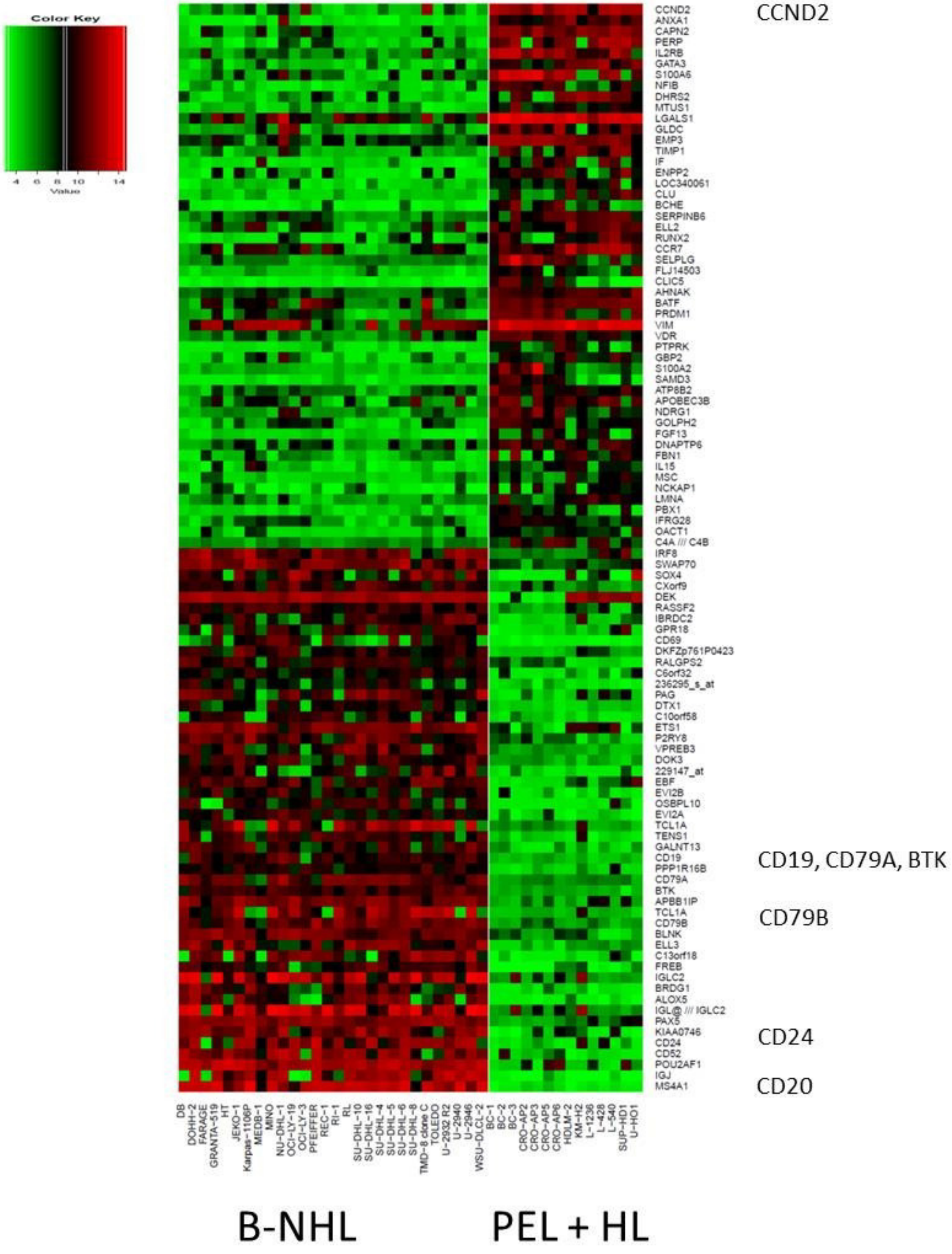

Supplementary Figure 2. Expression of genes in PEL and HL. Expression array analysis showed that PEL and HL cell lines share a number of up- and downregulated genes (e.g. *CCND2* upregulated, *BTK* and *CD19* repressed) as opposed to non-PEL B-NHL cell lines.

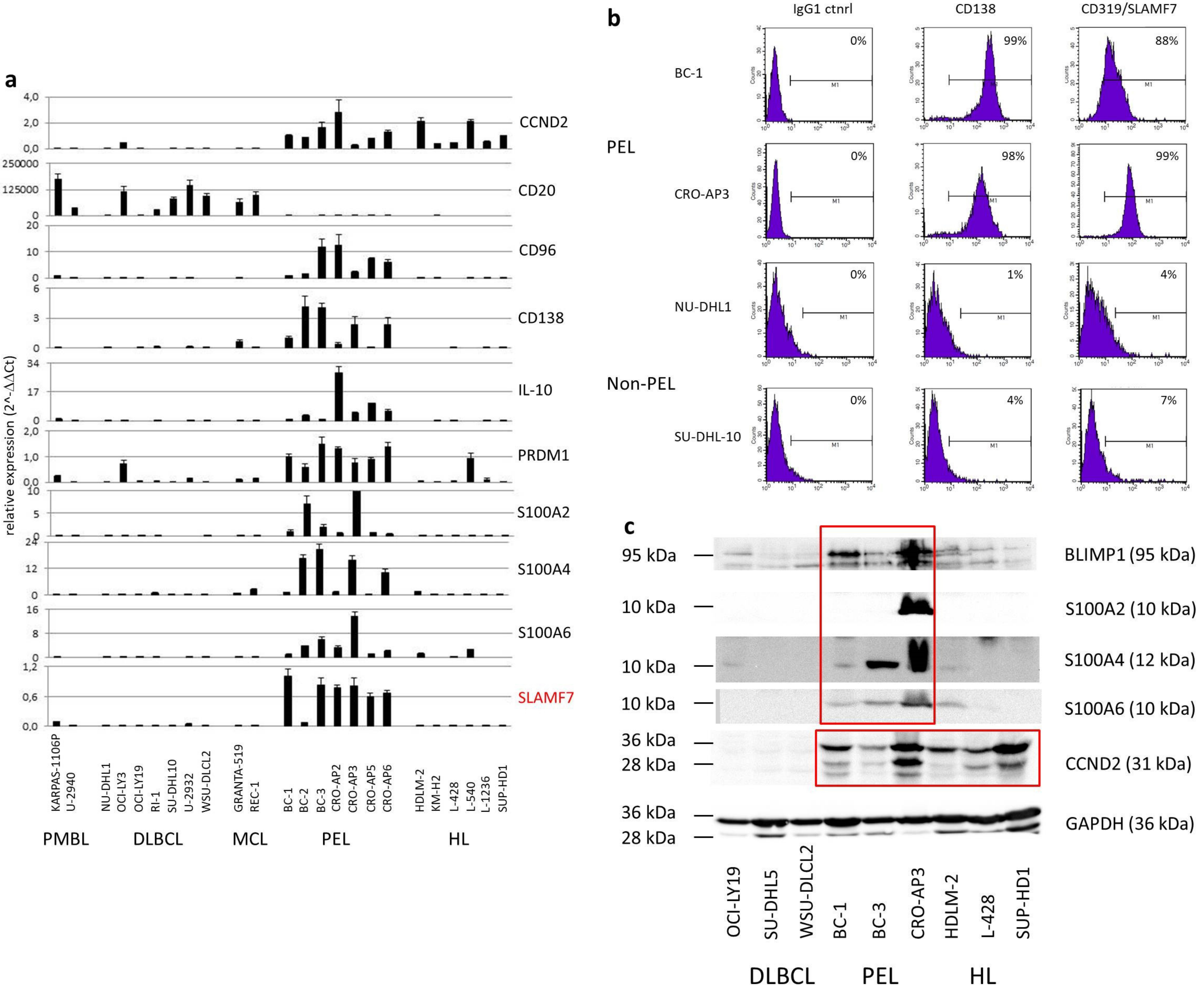

Supplementary Figure 3. PEL-characteristic gene expression pattern. A) RQ-PCR, B) flow-cytometry and C) Western blot analysis confirmed PEL-specific expression of *IL-10, PRDM1, S100A2, S100A4, S100A6* and *SLAMF7.* Not shown in the Figure are RQ values for cell line L-363 (*SLAMF7* RQ = 3.4), BC-2 (*S100A2* RQ = 8.3) and CRO-AP3 (*S100A2* RQ = 120).

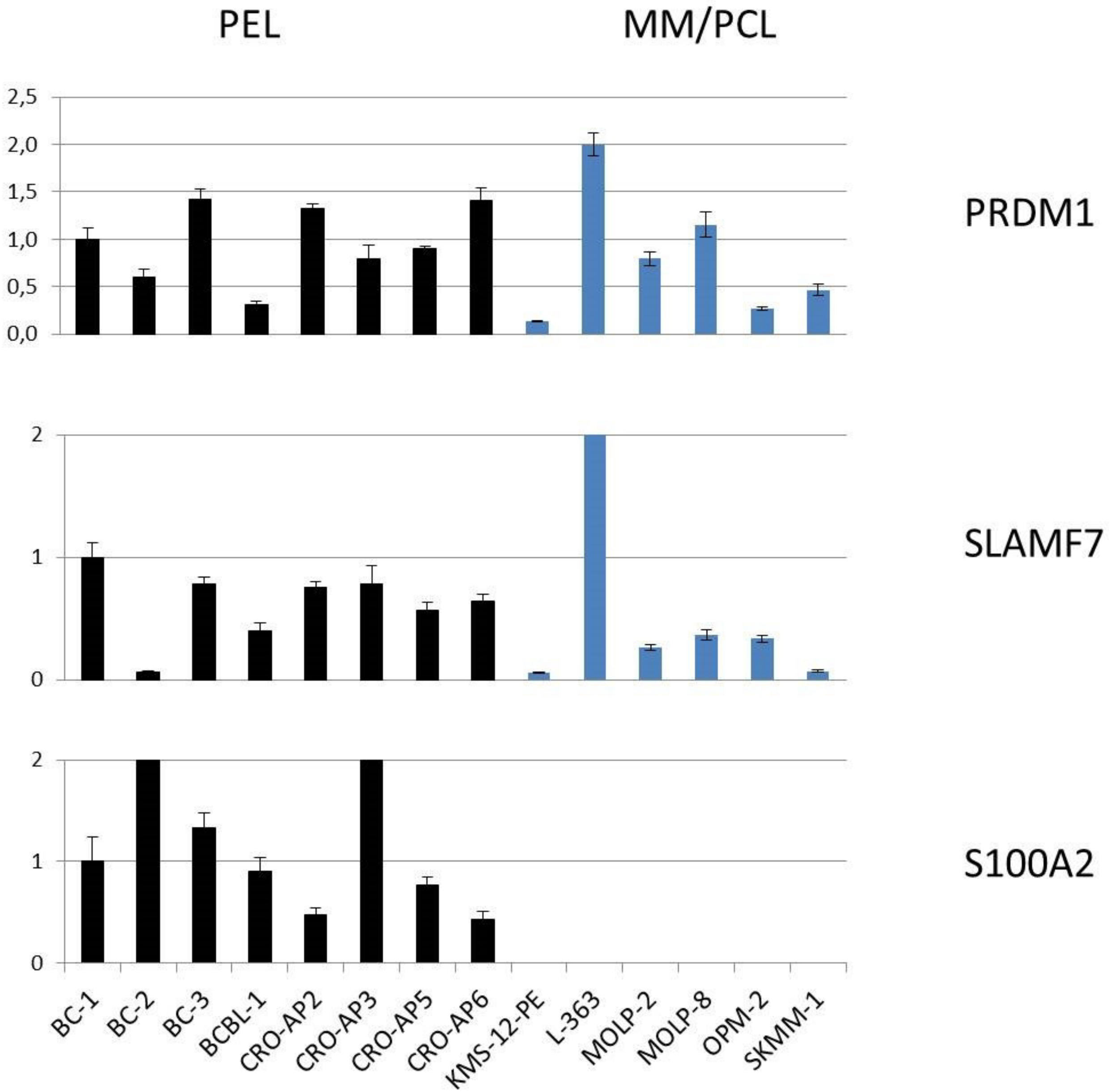

Supplementary Figure 4. *SLAMF7* in PEL vs MM/PCL. RT-PCR analysis confirming comparable levels of *SLAMF7* mRNA in primary effusion lymphoma (PEL) and multiple myeloma (MM) / plasma cell leukemia (PCL) cell lines.

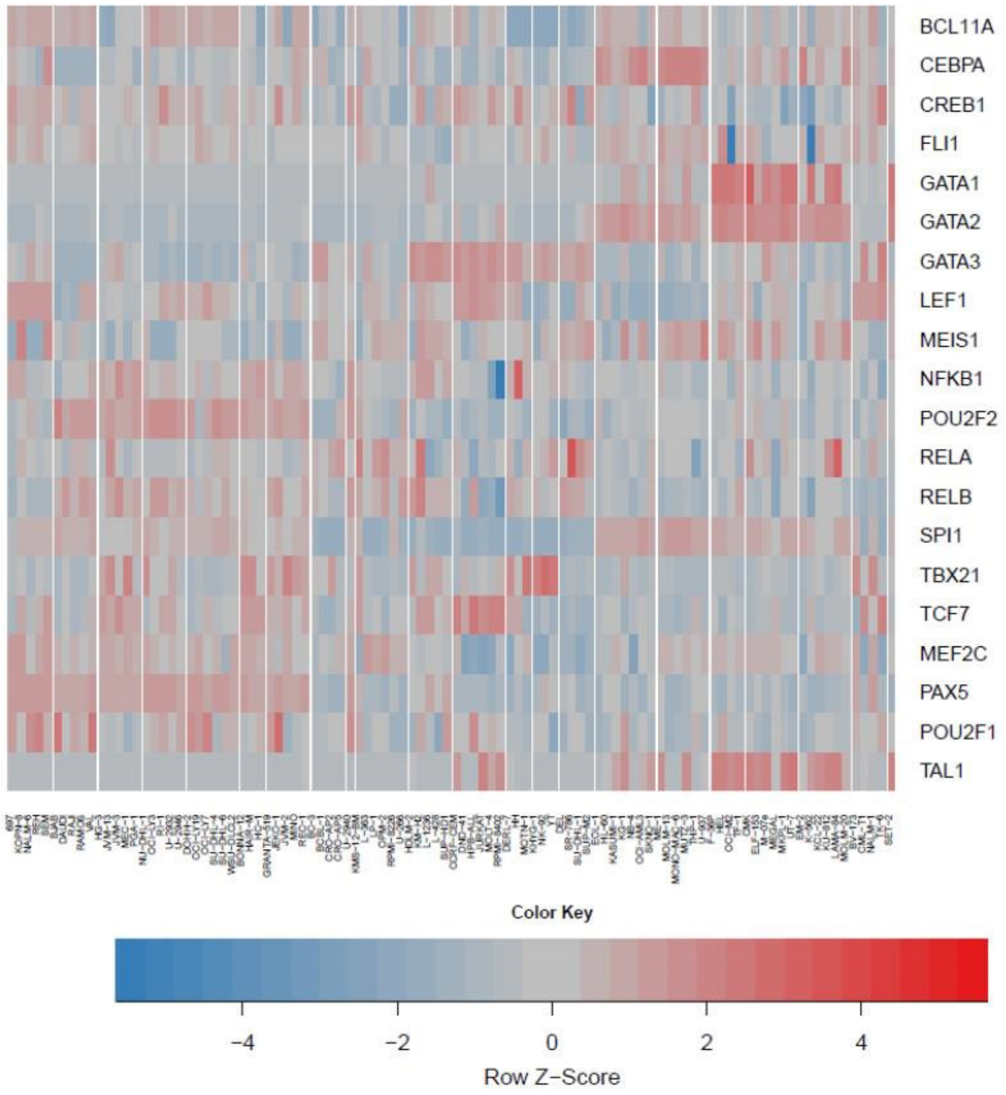

Supplementary Figure 5. Relative gene expression of hematopoietic TFs across LL-100 cell lines. Heatmap of gene expression levels according to RNA-seq data from each cell line. Each column represents a single cell line which are grouped according to entities. The color code depicts the relative gene expression (logCPM) for each row-wise normalized TF.

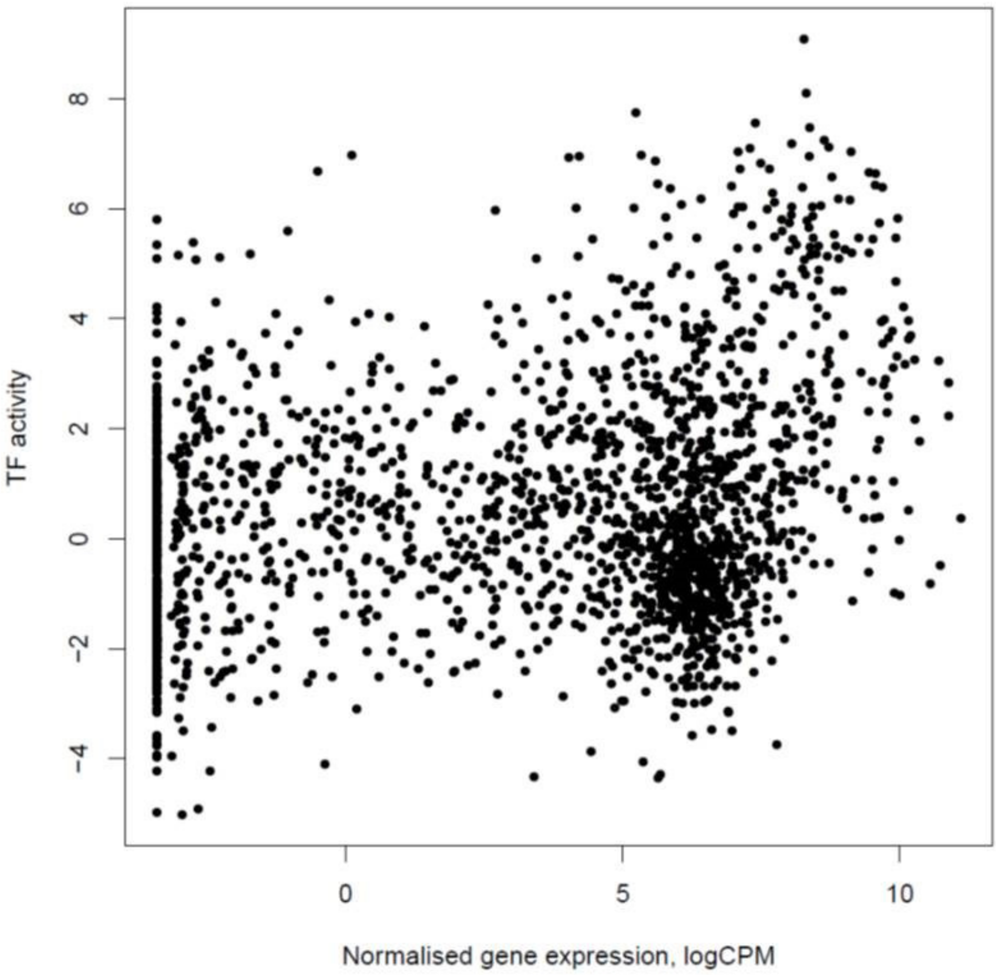

Supplementary Figure 6. Lack of correlation between gene expression levels of hematopoietic TFs and their transcriptional activity according to CTFR activity scores on global level. Scatter plot depicting normalized gene expression (logCPM) for each of the selected hematopoietic TFs in each cell line plotted against its transcriptional activity as computed via VIPER based on DoRothEA interactions.

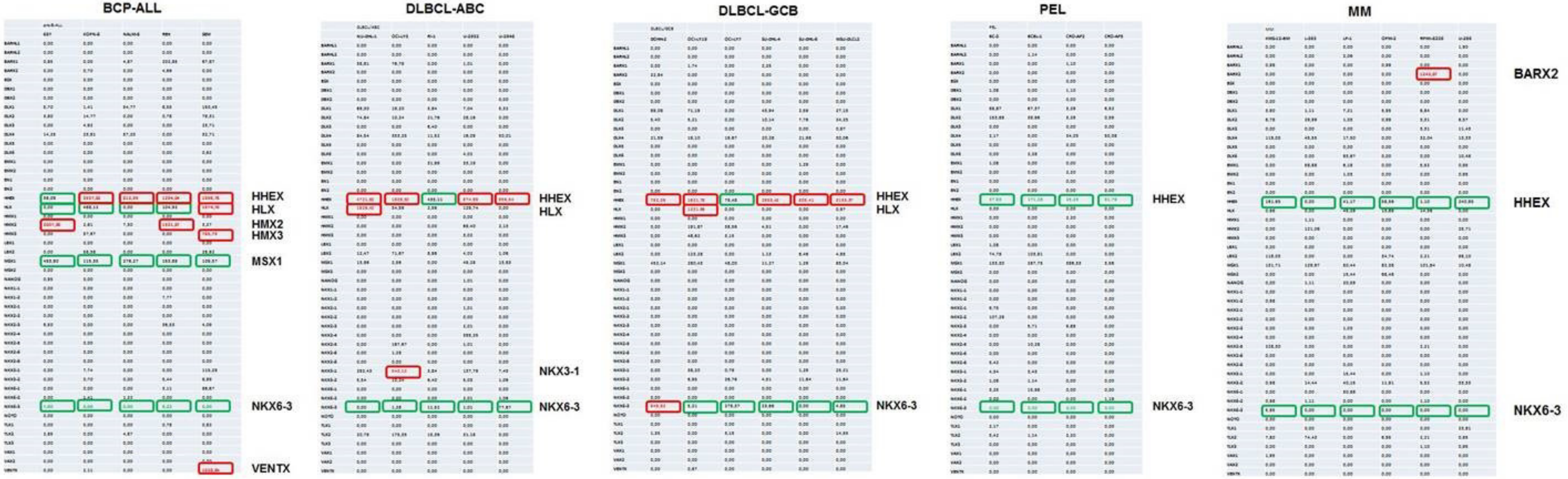

Supplementary Figure 7. Expression data of NKL homeobox genes in cell lines derived from BCP-ALL, DLBCL, PEL and MM. The indicated tables show expression levels of 48 NKL homeobox genes (from left to right) in five BCP-ALL cell lines, in five cell lines derived from DLBCL-ABC, in six cell lines derived from DLBCL-GCB, four PEL cell lines, and in four MM cell lines. The listed values are normalized counts (reads) obtained by DESeq2 calculation. Overexpressed or downregulated gene activities are highlighted by red or green boxes, respectively.

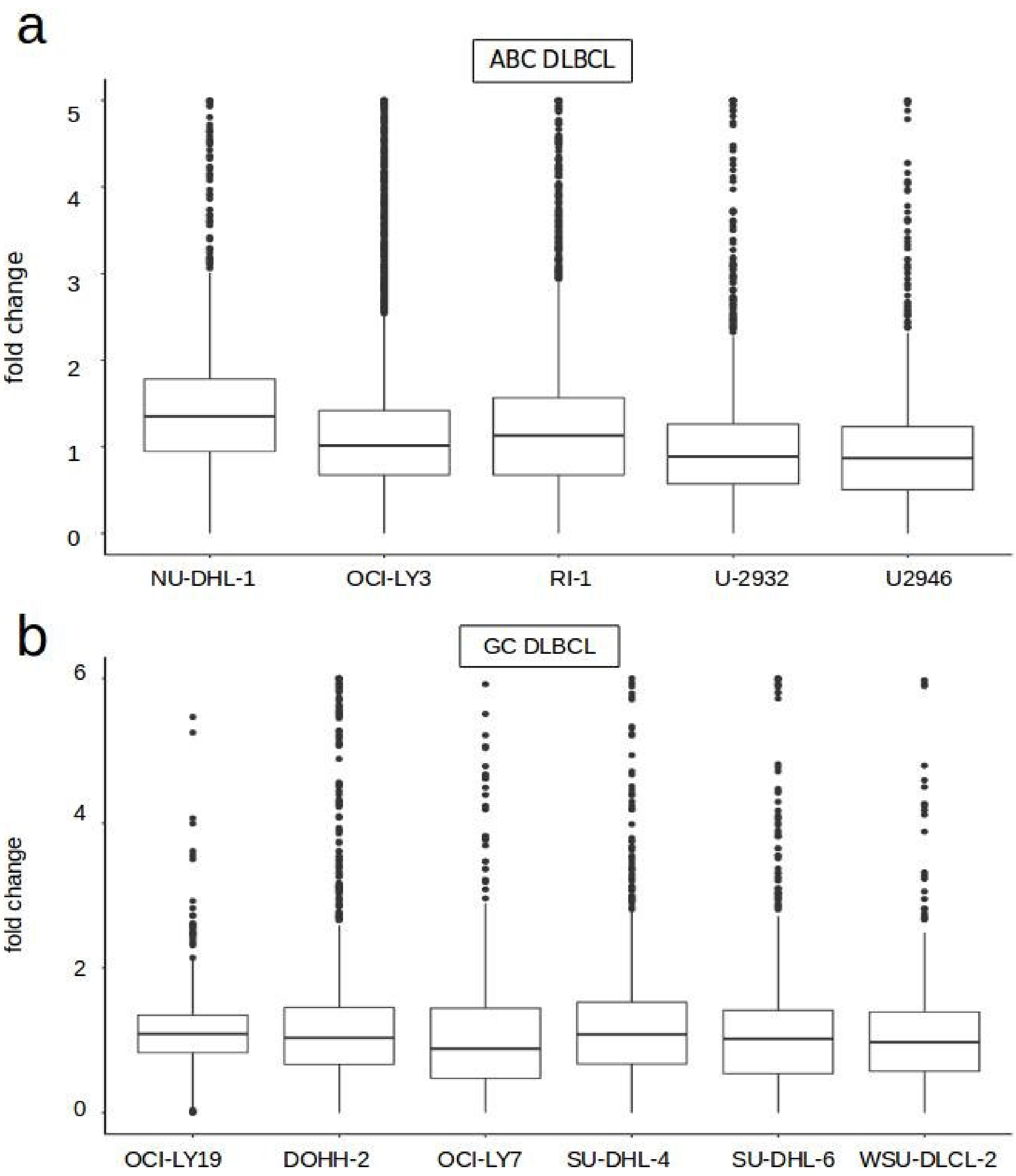

Supplementary Figure 8. Expressional changes of genes within regions of CNAs. Shown is the fold change for each gene within CNAs for the resepctive cell line calculated about the median from all ABC DLBCL (a) or GC DLBCL (b) cell lines.

LIMS1

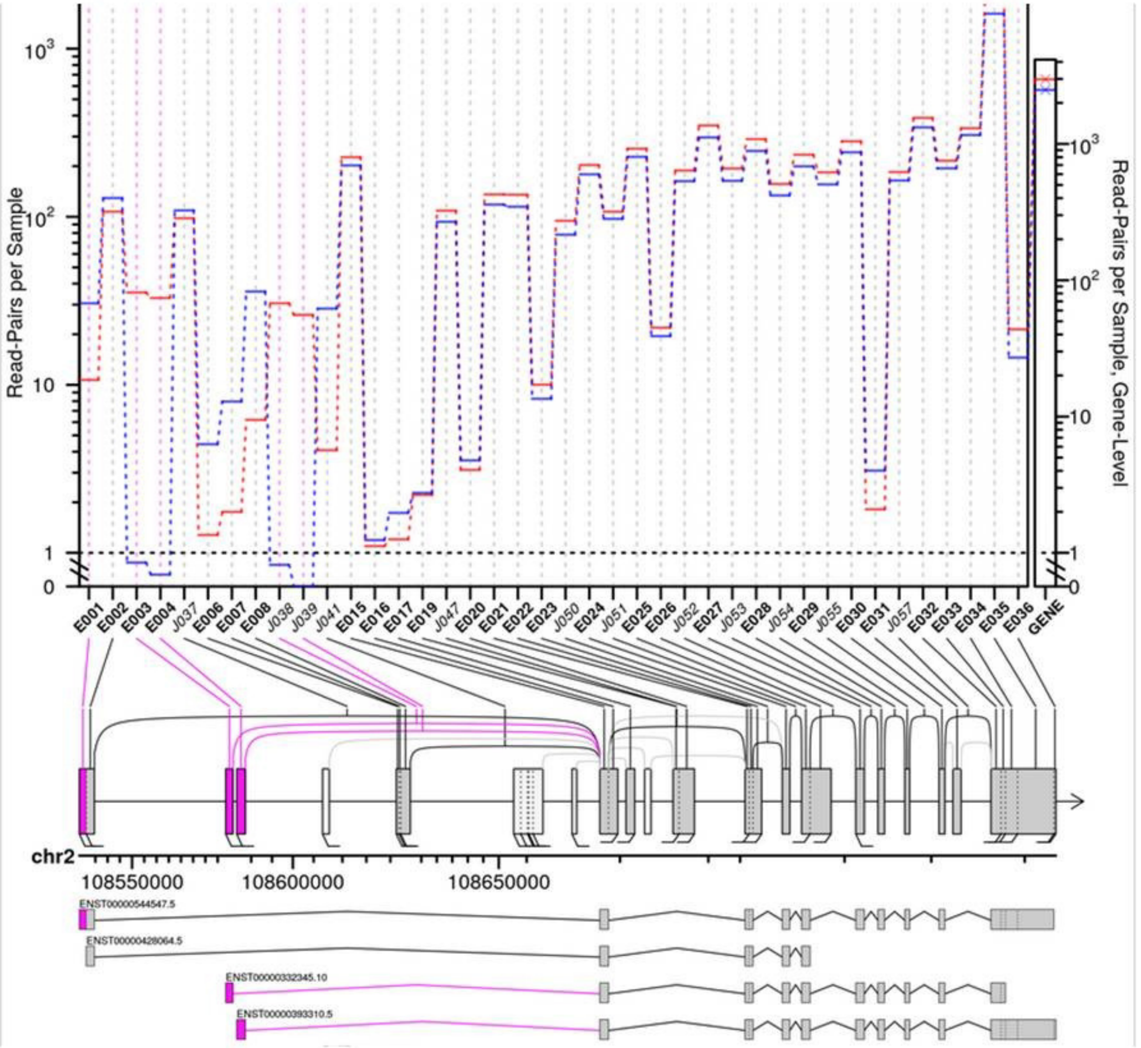

Supplementary Figure 9. Mean normalized coverage of *LIMS1* in myeloid vs T-cell lines. RNA-seq data show expression of two N-terminal LIMS1 variants in myeloid cell lines (red line), only one in T-cell lines (blue line). Myeloid cell lines: HL-60, KASUMI-1, KG-1, ME-1, MOLM-13, MONO-MAC-6, MUTZ-3, NB-4, OCI-AML-3, THP-1; T-cell lines: CCRF-CEM, DERL-7, DND-41, HH, HPB-ALL, JURKAT, MOLT-4, MOTN-1, RPMI-8402.

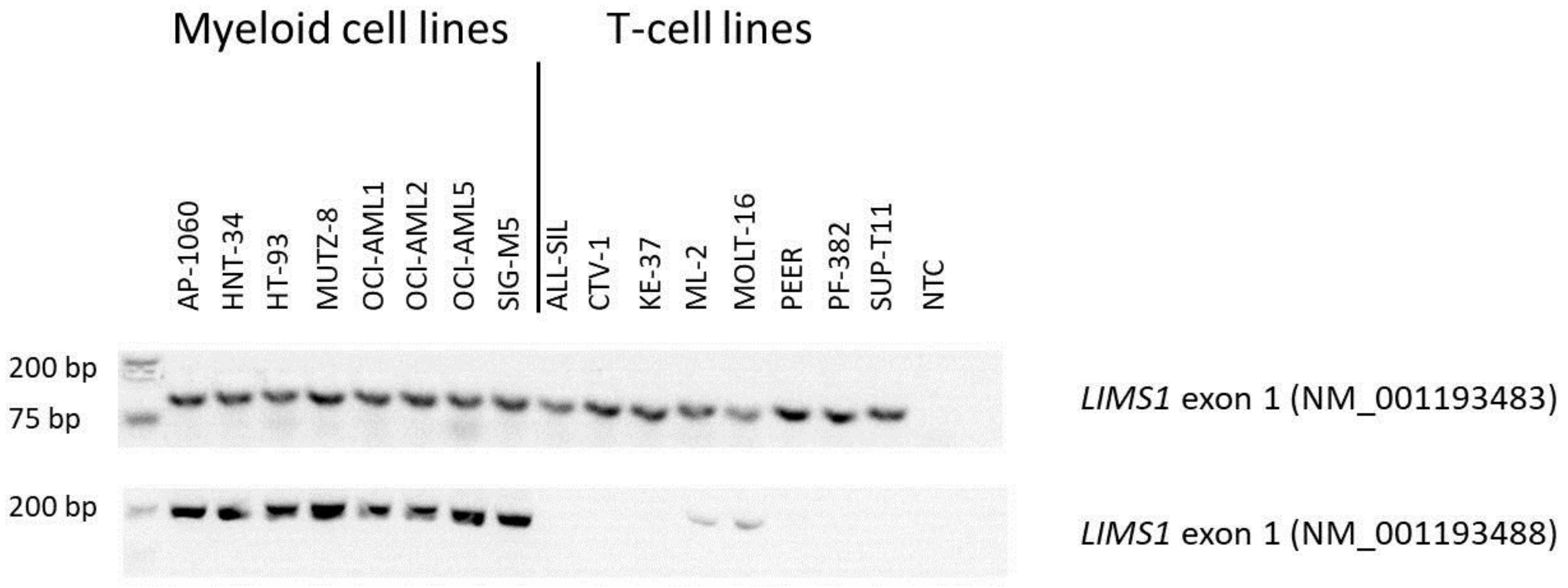

Supplementary Figure 10. Expression of N-terminal *LIMS1* exons in myeloid and T-cell lines. RT-PCR analysis of validation cohort.
